# Epigenetic Regulation of Learning and Memory by *Drosophila* EHMT/G9a

**DOI:** 10.1371/journal.pbio.1000569

**Published:** 2011-01-04

**Authors:** Jamie M. Kramer, Korinna Kochinke, Merel A. W. Oortveld, Hendrik Marks, Daniela Kramer, Eiko K. de Jong, Zoltan Asztalos, J. Timothy Westwood, Hendrik G. Stunnenberg, Marla B. Sokolowski, Krystyna Keleman, Huiqing Zhou, Hans van Bokhoven, Annette Schenck

**Affiliations:** 1 Department of Human Genetics, Nijmegen Centre for Molecular Life Sciences, Donders Institute for Brain, Cognition and Behaviour, Radboud University Nijmegen Medical Centre, Nijmegen, The Netherlands; 2 Radboud University Nijmegen, Department of Molecular Biology, Nijmegen Centre for Molecular Life Sciences, Faculty of Science, Nijmegen, The Netherlands; 3 Aktogen Ltd., Department of Genetics, University of Cambridge, Cambridge, United Kingdom; 4 Institute of Biochemistry, Biological Research Center of Hungarian Academy of Sciences, Szeged, Hungary; 5 Department of Biology, University of Toronto, Ontario, Canada; 6 Institute of Molecular Pathology, Vienna, Austria; 7 Department of Cognitive Neurosciences, Donders Institute for Brain, Cognition and Behavior; Radboud University Nijmegen Medical Centre, Nijmegen, The Netherlands; North Carolina State University, United States of America

## Abstract

The epigenetic modification of chromatin structure and its effect on complex neuronal processes like learning and memory is an emerging field in neuroscience. However, little is known about the “writers” of the neuronal epigenome and how they lay down the basis for proper cognition. Here, we have dissected the neuronal function of the *Drosophila* euchromatin histone methyltransferase (EHMT), a member of a conserved protein family that methylates histone 3 at lysine 9 (H3K9). EHMT is widely expressed in the nervous system and other tissues, yet EHMT mutant flies are viable. Neurodevelopmental and behavioral analyses identified EHMT as a regulator of peripheral dendrite development, larval locomotor behavior, non-associative learning, and courtship memory. The requirement for EHMT in memory was mapped to 7B-Gal4 positive cells, which are, in adult brains, predominantly mushroom body neurons. Moreover, memory was restored by EHMT re-expression during adulthood, indicating that cognitive defects are reversible in EHMT mutants. To uncover the underlying molecular mechanisms, we generated genome-wide H3K9 dimethylation profiles by ChIP-seq. Loss of H3K9 dimethylation in EHMT mutants occurs at 5% of the euchromatic genome and is enriched at the 5′ and 3′ ends of distinct classes of genes that control neuronal and behavioral processes that are corrupted in EHMT mutants. Our study identifies *Drosophila* EHMT as a key regulator of cognition that orchestrates an epigenetic program featuring classic learning and memory genes. Our findings are relevant to the pathophysiological mechanisms underlying Kleefstra Syndrome, a severe form of intellectual disability caused by mutations in human *EHMT1*, and have potential therapeutic implications. Our work thus provides novel insights into the epigenetic control of cognition in health and disease.

## Abstract 2

Behavioral phenotyping and genome-wide profiling of the histone modifier EHMT in *Drosophila* reveals a mechanism through which an epigenetic writer may control cognition.

## Author Summary

Epigenetic regulators can affect gene transcription through modification of DNA and histones, which together form chromatin. The importance of such regulators for cognition is increasingly appreciated, but only few key factors have been identified so far. Excellent candidates are histone modifiers that are involved in intellectual disability, such as EHMT1, implicated in Kleefstra Syndrome. Here, we characterized the neuronal function of EHMT in *Drosophila*. Flies that lack EHMT are viable but show highly selective defects in specific aspects of neuronal development and function, including learning and memory. Genome-wide analysis of EHMT-mediated histone methylation revealed that EHMT targets the majority of all currently known *Drosophila* learning and memory genes. It also targets genes known to be involved in the other aspects of behavior and neuronal development that are compromised in EHMT mutants. Remarkably, EHMT mutant memory deficits can be reversed in adulthood, suggesting that epigenetic influences on cognition are not always permanent. Our results provide novel insights into the epigenetic control of cognition in health and disease.

## Introduction

Modification of chromatin structure regulates many aspects of cell and developmental biology. Epigenetic regulators are known to affect complex neuronal processes such as learning and memory [1]–[3] and contribute significantly to the occurrence of cognitive disorders, such as schizophrenia and intellectual disability (previously referred to as mental retardation) [4],[5]. However, little is known about the “writers” of the neuronal epigenome that lay down the basis for proper cognition. Are these chromatin writers required to safeguard neuronal homeostasis/fitness by influencing the expression of a large and heterogeneous group of genes, such as house-keeping and non-neuronal genes? Or do they lay down specific epigenetic programs to regulate neuronal genes that are directly involved in determination of connectivity, plasticity, learning, and memory?

The euchromatin histone methyltransferases (EHMTs) are a family of evolutionarily conserved proteins that write part of the epigenetic code through methylation of histone 3 at lysine 9 (H3K9) [6]–[10]. In mammals, two EHMT paralogs exist, EHMT1/GLP and EHMT2/G9a. Heterozygous mutations or deletions of the human *EHMT1* gene cause Kleefstra Syndrome (OMIM #610253), a neurodevelopmental disorder that is characterized by autistic-like features and severe intellectual disability [11]–[14]. Studies in mice have shown that Ehmt1/GLP and Ehmt2/G9a form a heterodimeric complex [8], and that loss of either protein resulted in nearly identical phenotypes, such as early embryonic lethality, reduced H3K9 dimethylation (H3K9me2), and inappropriate gene transcription [7],[8]. Furthermore, mice with neuronal ablation of Ehmt1/GLP and Ehmt2/G9a in adulthood show defects in fear conditioned learning. A gene expression study in different brain areas of these mice has led to the suggestion that EHMT proteins act as repressors of non-neuronal genes in neuronal tissues [15]. Here, by using classic *Drosophila* genetics, extensive neurodevelopmental and behavioral phenotyping, expression profiling, and genome-wide mapping of EHMT target loci by H3K9me2 profiling, we uncover EHMT as a key epigenetic regulator of neuronal genes and processes.

## Results

### EHMT/G9a Is the Solitary *Drosophila* Ortholog of EHMT1 and EHMT2/G9a

**Figure 1:**
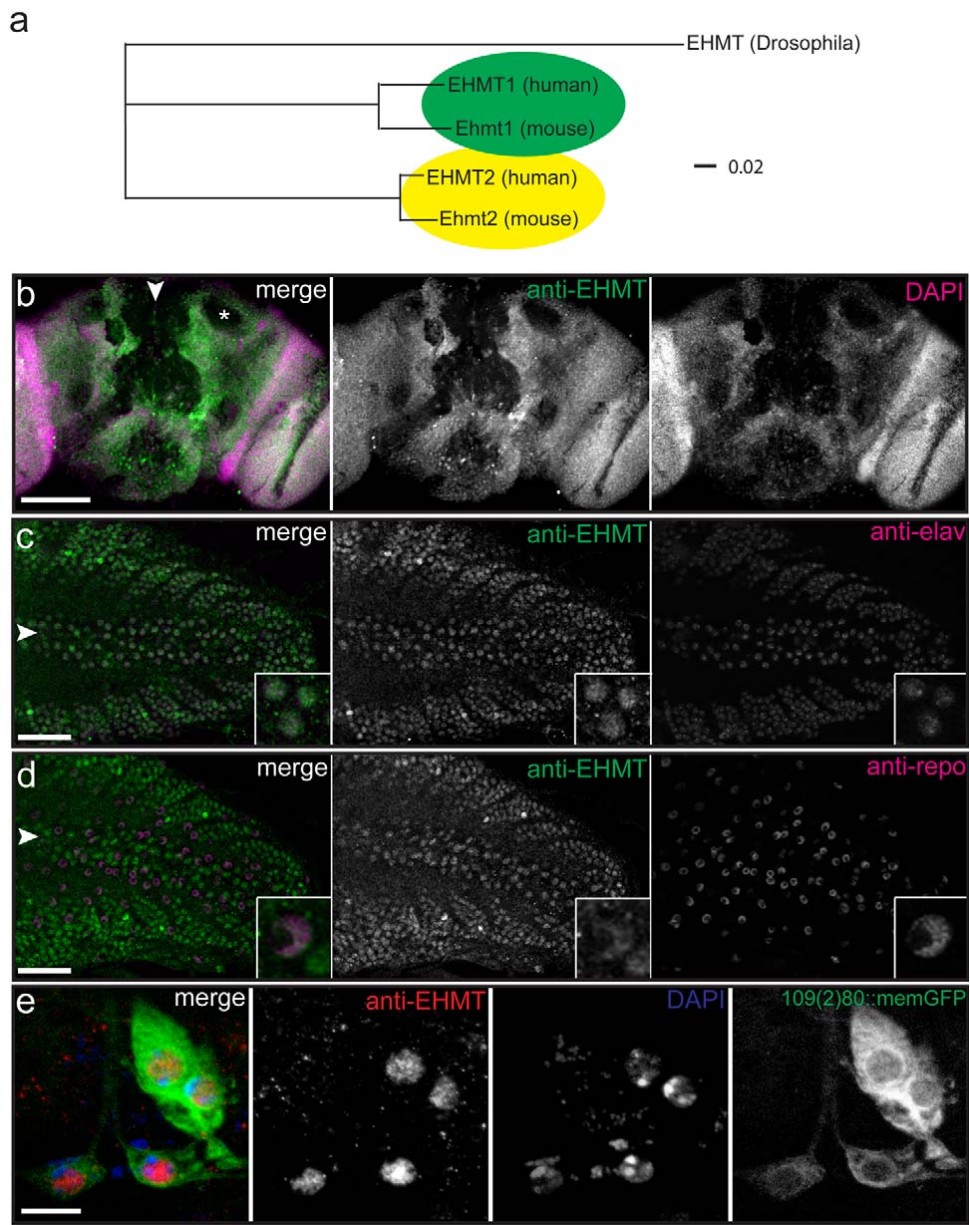
*EHMT* phylogeny and expression in the nervous system. (a) A phylogenetic tree generated using the neighbor joining method showing the evolutionary relationship between *Drosophila* EHMT and the mouse and human orthologs of EHMT1 and EHMT2/G9a. Analysis was performed with the Vector NTI software (Invitrogen). The scale bar indicates phylogenetic distance. (b) Frontal view of an adult *Drosophila* brain stained with an antibody to EHMT (green) and counterstained with DAPI (magenta). EHMT is widely expressed but excluded from neuropilar regions such as the mushroom body calyx (asterisk in merge panel). (c–d) Horizontal view of a third instar larval ventral nerve cord (vnc) stained with an anti-EHMT antibody (green) and counterstained with (c) anti-elav (magenta) to highlight neuronal nuclei or with (d) anti-repo (magenta) labeling glial nuclei. EHMT colocalizes with elav and repo but appears to be expressed at a lower level in glia. (e) The ventral cluster of multiple dendrite neurons of the larval body wall labeled with memGFP (green) using the *109(2)80-Gal4* driver and stained with anti-EHMT (red) and DAPI (blue). Arrowheads point to the midline of the brain (in b) and vnc (in c, d). Scale bars represent 100 µm in (b), 50 µm in (c–d), and 10 µm in (e). Anterior is to the left (c–e).

We set out to study EHMT**/**G9a in *Drosophila*. In contrast to mammals, which have two *EHMT* genes, flies possess a single ortholog [6],[10] that we will subsequently refer to as EHMT throughout our manuscript. Phylogenetic analysis of the EHMT protein family in *Drosophila*, human, and mouse shows that *Drosophila* EHMT is equally similar to both EHMT1 and EHMT2/G9a (Figure 1a).

### EHMT Is Expressed in the Fly Nervous System

**Figure 2:**
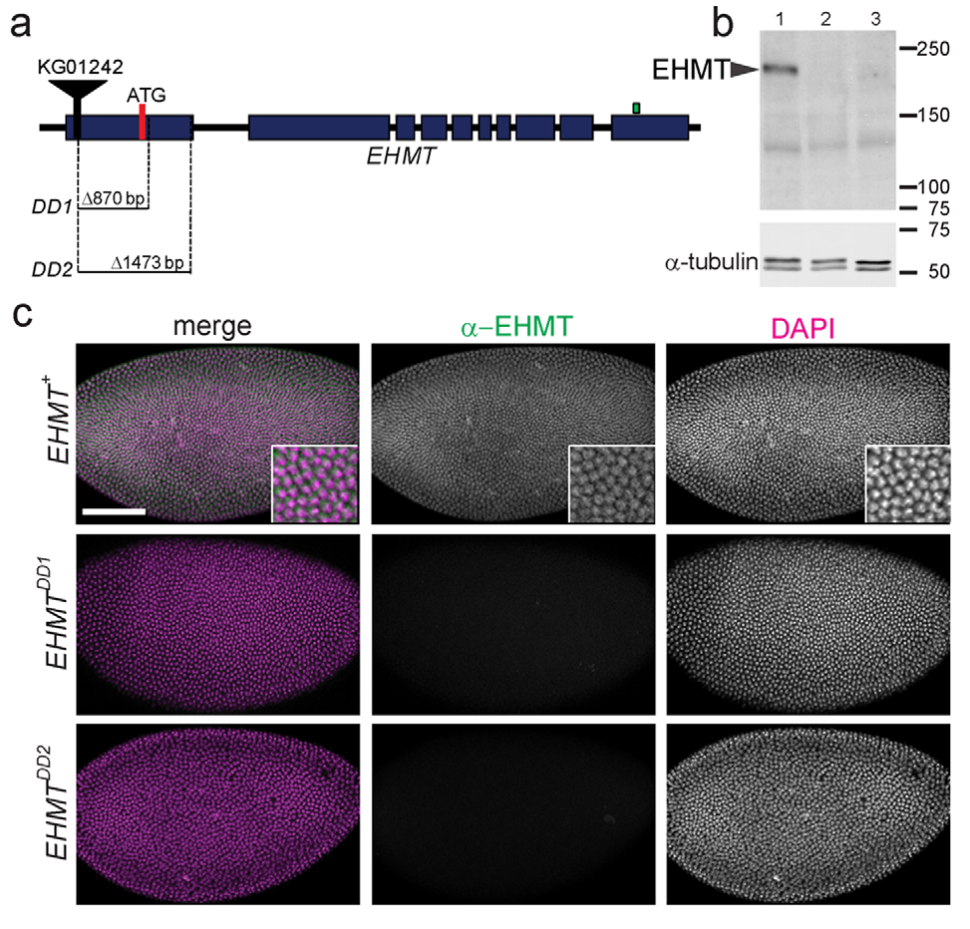
Molecular characterization of *EHMT* alleles. (a) Schematic depiction of the genomic region containing *EHMT* and the location of the *EHMT* deletions. Blue boxes indicate *EHMT* exons. The red bar shows the location of the predicted translational start site. The black bar and triangle indicate the location of the P-element insertion *KG01242*. The green bar marks the epitope of the EHMT antibody. The size and location of the *EHMT* deletions *DD1* and *DD2* (downstream deletions 1 and 2) is indicated. (b) Western blot using anti-EHMT and embryonic protein extracts from *EHMT* ^+^ (lane 1), *EHMT^DD1^* (lane 2), and *EHMT^DD2^* (lane 3).The EHMT band (arrowhead) is positioned in accordance with a predicted size of 180 kDa. This band is absent in the deletion lines and no extra bands were observed. α-tubulin was used as loading control. (c) *EHMT* ^+^, *EHMT^DD1^*, and *EHMT^DD2^* embryos at blastoderm stage, stained with anti-EHMT (green) and DAPI (magenta). EHMT staining is nuclear in wild-type and absent in mutant *EHMT* embryos. Scale bar is 100 µm.

We first examined expression and subcellular localization of the EHMT protein in the fly nervous system using an anti-EHMT antibody [10]. In the adult brain EHMT staining is widely abundant, in a pattern resembling nuclear DAPI staining (Figure 1b). Analysis at single cell resolution in the ventral nerve cord of third instar larvae demonstrates that EHMT is localized in the nuclei of neurons as revealed by colocalization with the neuronal nuclear marker elav (Figure 1c) [16]. Weaker EHMT staining colocalized with repo (Figure 1d), a nuclear glial marker [17]. EHMT staining was also observed in the nuclei of the larval multiple dendrite (md) sensory neurons of the peripheral nervous system labeled using the *109(2)80-Gal4* driver [18] to express memGFP (Figure 1e). In addition, EHMT staining was observed at low levels in non-neuronal tissues such as the muscle and epidermis (Figure S1c). The anti-EHMT immunolabeling is specific, as it is absent in *EHMT* mutant embryos, md neurons, adult brains, and larval body-walls (Figures 2c and S1). These data reveal that EHMT is widely expressed in the *Drosophila* nervous system.

### Generation of *EHMT* Mutant Flies

In order to uncover the functions of EHMT we generated deletions in the *EHMT* gene by excision of a P-element, *KG01242*, located in the 5′ UTR. We screened 80 independent excision lines and identified two downstream deletions (DD) resulting from imperfect excisions of *KG01242*. Both deletion strains are viable to adulthood, which is consistent with a viable EHMT knock-out allele generated by homologous recombination in *Drosophila* [19]. *EHMT^DD1^* and *EHMT^DD2^* lack 870 and 1473 base pairs of DNA downstream of the original P-element insertion site, respectively, including the EHMT translational start site (Figure 2a). We also isolated a precise transposon excision line that represents the same genetic background as our deletion lines and served as a control in all subsequent experiments (referred to as *EHMT^+^*). Western blot analysis revealed a band of the expected size (180 kDa) in *EHMT* ^+^ embryonic protein extracts, which was absent in extracts from both deletion lines (Figure 2b). No extra bands were detected by the C-terminally directed EHMT antibody [10] that would point to expression of an N-terminally truncated protein. EHMT protein was also undetectable by immunohistochemistry in *EHMT* mutant embryos, md neurons, and adult brains, while showing a nuclear staining pattern in *EHMT* ^+^ animals (Figures 1, 2c, and S1). Expression of the neighbor gene, *CG3038*, was not affected by the deletions (Figure S2). These data show that *EHMT^DD1^* and *EHMT^DD2^* are strong and specific loss of function mutants, most likely complete null alleles.

### *EHMT* Regulates Dendrite Branching in Type 4 Multiple Dendrite Neurons

Next, we examined several aspects of neuronal development in *EHMT* mutant flies. Analysis of adult mushroom body architecture, synaptic morphology of the larval neuromuscular junction, and adult photoreceptor function (assessed by electroretinography) (Figure S3) as well as analysis of embryonic nervous system integrity (unpublished data) did not reveal significant differences in mutant versus control conditions, indicating that general nervous system development and neuronal function is not affected.

**Figure 3:**
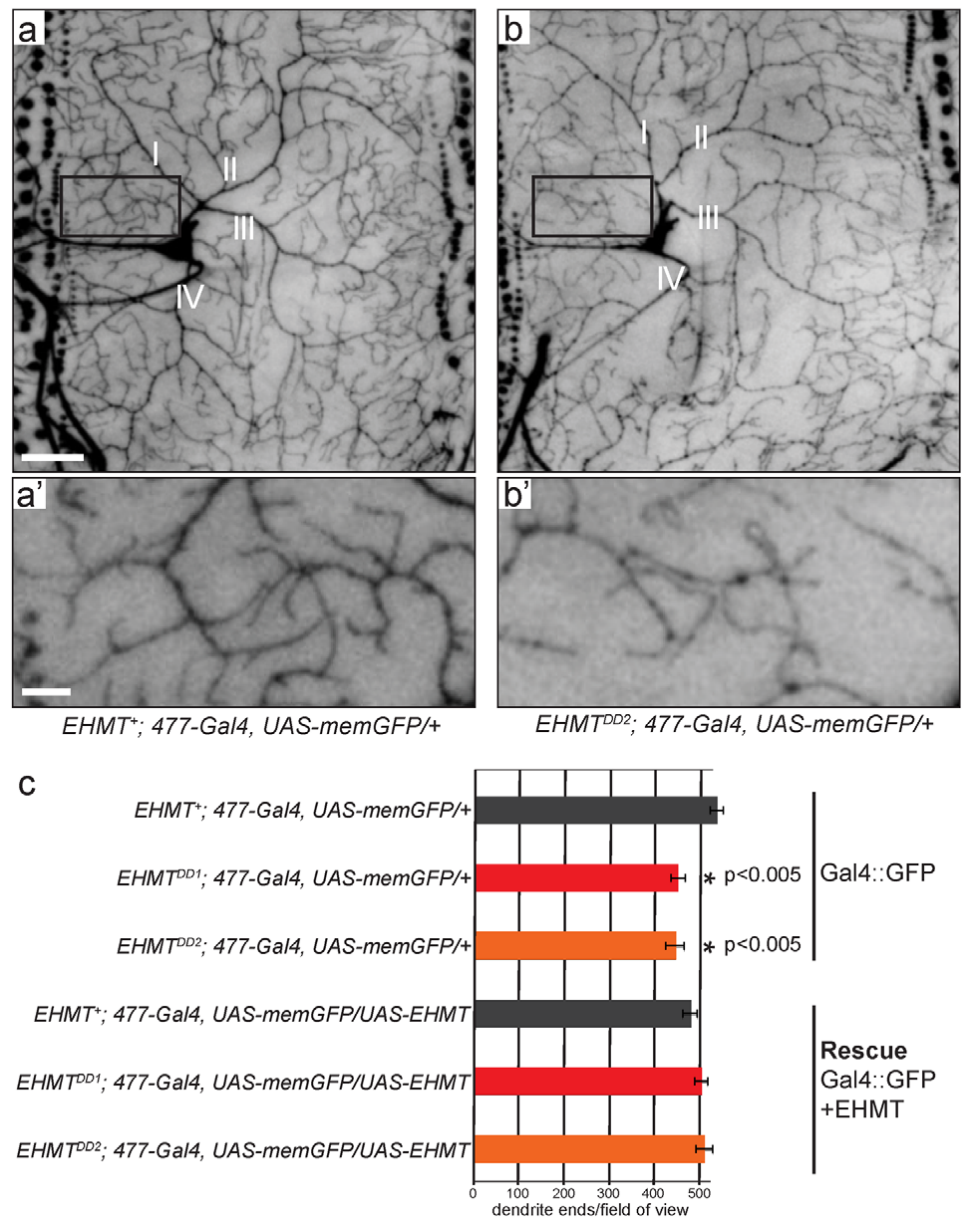
*EHMT* regulates dendrite branching. (a, b) Representative confocal images of ventral type 4 md neurons from (a) *EHMT* ^+^ and (b) *EHMT^DD2^* third instar larvae labeled by expression of *UAS-memGFP* using the *477-Gal4* driver. I, II, III, and IV label the primary dendrite branches, which are conserved in all neurons examined. Scale bars represent 50 µm. (a', b') High magnifications of neurons shown in (a) and (b), respectively. Scale bars represent 10 µm. (c) Quantitative evaluation of dendrite ends per field of view, as shown in (a–b). Note that dendrite branching was significantly reduced in *EHMT* mutants. 477*-Gal4* driven expression of *UAS-EHMT* caused a non-significant reduction of dendrite branches in the *EHMT* ^+^ background (black bars) and restored the number of dendrite ends in *EHMT^DD1^* (red bars) and *EHMT^DD2^* (orange bars). Error bars represent standard error of the mean. Complete genotypes are indicated. Sample sizes were *n* = 19, 20, 18 for the mutant conditions and 9, 8, and 11 for the rescue experiment, respectively, in *EHMT* ^+^, *EHMT^DD1^*, and *EHMT^DD2^*. (*) represents a significant difference to *EHMT^+^; 477-Gal4, UAS-memGFP/+* (*p*<0.05, one-way ANOVA and Bonferroni tests).

Loss of *EHMT* did however result in altered dendrite development in multidendrite (md) neurons, which are sensory neurons that tile the larval body wall. We specifically examined dendritic arbors of type 4 md neurons, which were highlighted using the *477-Gal4* driver [20] and the *UAS-Gal4* system [21] (Figure 3a and 3b). These neurons are highly stereotyped in their number, position, and morphology, thus allowing for quantitative analysis of dendritic arbors of identical neurons in different animals and genotypes. While the basic organization of these arbors is maintained in *EHMT* mutants (primary branches labeled I, II, III, and IV in Figure 3a and 3b), reduction of higher order branching resulted in dendritic fields of appreciably reduced complexity (Figure 3a, 3a' versus 3b, 3b'). We quantitatively assessed this defect by counting the number of dendrite ends per standardized field of view in stacked confocal images. This analysis confirmed that *EHMT^DD1^* and *EHMT^DD2^* had a consistent and statistically significant decrease in the total number of dendrite ends, showing 16 and 17.5 percent reduction, respectively, when compared to *EHMT^+^* (Figure 3c).

To address whether this phenotype results cell-autonomously from *EHMT* deficiency in neurons, we generated *UAS-EHMT* transgenic flies and performed cell-specific rescue experiments (Figure 3c). Re-expression of *EHMT* in mutant type 4 md neurons (using *477-Gal4*) did indeed rescue dendrite branching towards wild-type levels (Figure 3c, red and orange bars). This reversal is specific, since expression of *EHMT* in the *EHMT* ^+^ genetic background did not increase branching (Figure 3c, black bars). Rather, *EHMT* overexpression appeared to reduce dendrite branching as compared to controls expressing Gal4 and GFP in the absence of *UAS-EHMT*, although this reduction was not statistically significant (Figure 3c, black bars). These data show that EHMT is cell-autonomously required in type 4 md neurons to establish normal dendrite complexity.

### EHMT Affects Larval Locomotory Behavior

**Figure 4:**
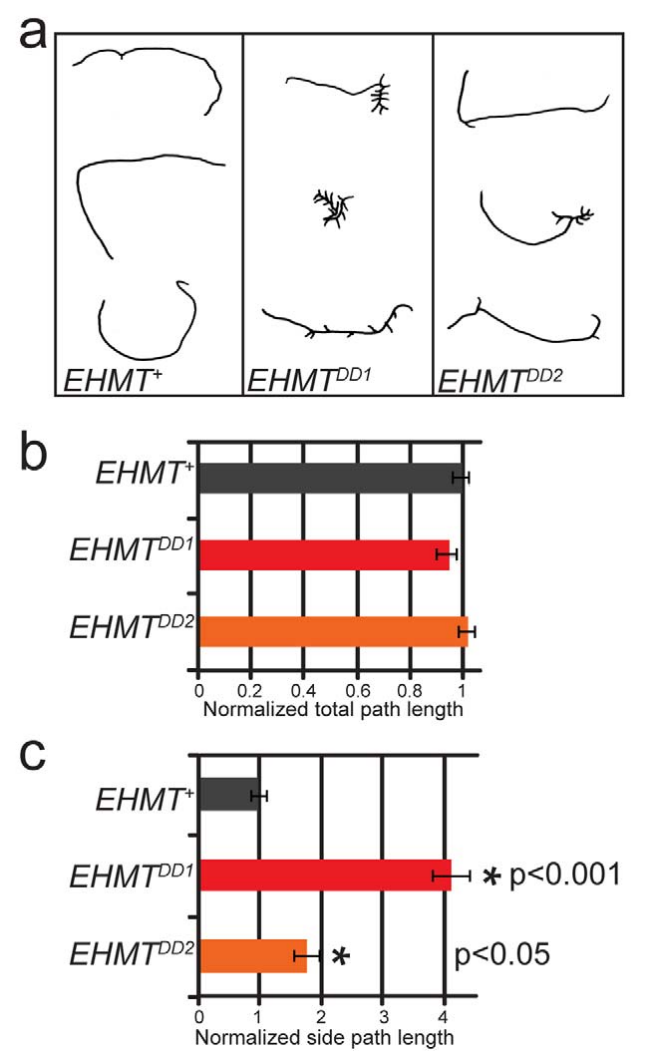
EHMT mutants show altered larval locomotory behavior. (a) Larval crawling paths from *EHMT* ^+^, *EHMT^DD1^*, and *EHMT^DD2^*. (b–c) Quantification of larval locomotory patterns revealed no significant difference in the (b) total path length but showed a significant increase in (c) side track length in both *EHMT^DD1^* (red bars) and *EHMT^DD2^* (orange bars). Data are normalized to EHMT^+^ and error bars represent the standard error of the mean. *N* = 149, 148, and 149 for *EHMT* ^+^, *EHMT^DD1^*, and *EHMT^DD2^*, respectively. (*) indicates a significant difference to *EHMT* ^+^ (*p*<0.05, Kruskal-Wallis and Mann-Whitney tests).

*Drosophila* md neurons are important in the regulation of larval locomotion behavior [22]–[24]. We therefore examined larval locomotory patterns during the early third instar using an established larval foraging assay (Figure 4) [25]. Larval crawling paths were analyzed for total path length over a 5 min period and for specific crawling patterns, such as branched versus straight paths. The total path length covered by foraging larvae was not different between mutants and *EHMT* ^+^ controls (Figure 4b), indicating that crawling ability is not hindered in these larvae. However, striking differences in larval locomotory patterns were observed between mutant and wild-type. Foraging *EHMT* mutant larvae often stop, retract, and turn, causing increased branching in their crawling paths (Figure 4a). Quantitative analysis of the length of the resulting side branches revealed an increase of approximately 4-fold and 2-fold, respectively, in *EHMT^DD1^* and *EHMT^DD2^* (Figure 4c). Thus, the dendrite phenotype of EHMT mutant larvae is associated with an altered crawling behavior. In contrast, other innate behaviors, such as adult phototaxis and negative geotaxis, were normal in *EHMT* mutants (Figure S4). To address whether the dendrite phenotype of type 4 md neurons alone is sufficient to cause the abnormal crawling pattern, we attempted to rescue this phenotype by re-expression of *UAS-EHMT* in type 4 md neurons using *477-Gal4*. This was not sufficient to restore normal larval locomotor behavior, indicating that dendritic defects in type 4 md neurons and abnormal locomotory behavior might arise independently.

### EHMT Regulates Habituation, a Form of Non-Associative Learning

**Figure 5:**
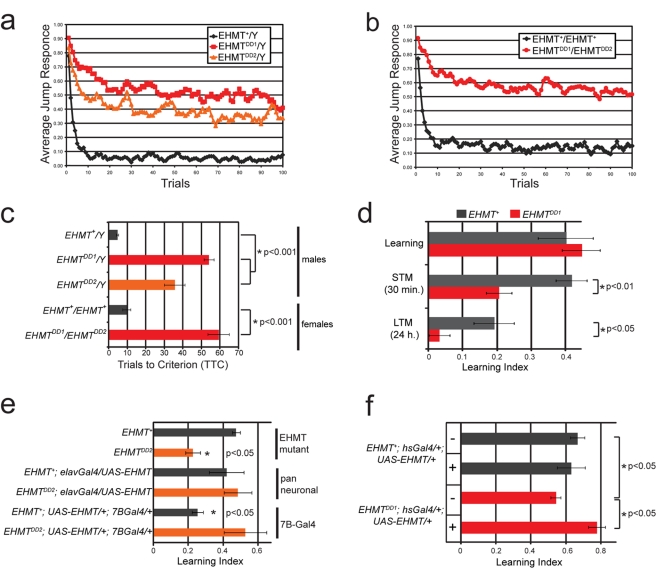
EHMT affects non-associative learning and courtship memory. (a–c) Jump reflex habituation. (a–b) Habituation was measured in (a) male flies of the genotypes *EHMT^+^/Y* (black diamonds), *EHMT^DD1^/Y* (red squares), and *EHMT^DD2^/Y* (orange triangles) and (b) female flies of the genotypes *EHMT^+^/EHMT^+^* (black diamonds) and *EHMT^DD1^/EHMT^DD2^* (red circles). Jumping was induced by repeated light-off pulses for 100 trials. (c) The mean number of trials to criterion was significantly higher for *EHMT* mutants (orange and red bars) than for *EHMT^+^* flies (black bars) after training with a 1 s inter-trial interval. (*) indicates a significant difference (*p*<0.001, one-way ANOVA and Bonferroni tests). (d–f) Learning and memory in the courtship conditioning paradigm. (d) The Learning Index (LI) of *EHMT^DD1^* males was normal at 0 min after training but was significantly reduced at 30 min after training (short term memory—STM) and 24 h after training (long term memory—LTM). (e) The LI of *EHMT^DD2^* males was also affected in short term memory and was rescued by expression of *EHMT* with the pan neuronal *elav-Gal4* driver and the *7B-Gal4* driver, which is predominantly expressed in the mushroom bodies in adult fly brains (orange bars). In the *EHMT^+^* background, expression of EHMT with *elav-Gal4* had no adverse effect, while expression with *7B-Gal4* caused a significantly decreased LI (black bars). (f) Short term memory was rescued in the *EHMT^DD1^* mutant background by induced expression of EHMT during adulthood, using the *hsGal4* driver. See Experimental Procedures for details on heat-shock conditions and courtship training. Error bars represent standard error of the mean. (*) indicates a significant difference (Kruskall-Wallis and Mann-Whitney tests).

Next, we analyzed the role of *Drosophila EHMT* in learning. Habituation is a form of non-associative learning where an initial response to a repeated stimulus gradually wanes [26]. In the light-off jump reflex habituation assay [27] flies were exposed to a sudden light-off pulse and measured for a jump response over the course of 100 trials with a 1 s inter-trial interval. Figure 5a and 5b show the proportion of flies that do show a jump response over the course of 100 trials. Hemizygous *EHMT* mutant males (genotypes: *EHMT^DD1^/Y* and *EHMT^DD2^/Y*) and transheterozygous *EHMT* mutant females (genotype: *EHMT^DD1^/EHMT^DD2^*) both displayed a drastically slower response decrement during the habituation procedure as compared to wild-type *EHMT^+^* flies (Figure 5a and 5b). Individual flies were deemed to have habituated when they failed to jump in five consecutive trials (no-jump criterion). Habituation was scored as the number of trials required to reach the no-jump criterion (trials to criterion). The mean number of trials to criterion for mutants, *EHMT^DD1^/Y*, *EHMT^DD2^/Y*, and *EHMT^DD1^/EHMT^DD2^*, was significantly higher (12-, 8-, and 6-fold, respectively) than for *EHMT^+^* wild-type flies (*p*<0.001) (Figure 5c). These experiments establish a role for EHMT in regulating non-associative learning.

### EHMT Is Required for Courtship Memory

Having established a role for EHMT in habituation, a simple learning process, we asked whether EHMT is also involved in more complex forms of learning and/or memory using the courtship conditioning paradigm. This assay is based on the conditioning of male courtship behavior by exposure to a non-receptive female, which in presence of normal learning and memory capacities results in suppression of courtship [28]. Male flies were paired with a non-receptive pre-mated female for appropriate time intervals (see Experimental Procedures) and tested for courtship suppression immediately following the training period, after 30 min or after 24 h, to assess learning, short-, and long-term memory, respectively. The mean Courtship Index (CI, the percentage of time spent on courtship during a 10 min interval) of trained males and of socially naïve males was assessed to calculate a Learning Index (LI), which is defined as the percent reduction in mean courtship activity in trained males compared to naïve males; LI  =  (CI_naive_ − CI_trained_)/CI_naive_. We found that EHMT mutant flies are perfectly capable of this form of learning, as they efficiently suppressed courtship immediately following the training period (Figure 5d). Strikingly, the Learning Index of *EHMT^DD1^* males was reduced by 50% at 30 min after training (STM-short term memory), and even more dramatically, to 17% of the wild-type value at 24 h after training (LTM-long term memory) (Figure 5d). These results indicate that EHMT is dispensable for courtship learning but necessary for both short- and long-term courtship memory.

### The Requirement for EHMT-Based Courtship Memory Maps to 7B-Gal4 Positive Neurons

To provide evidence for the specificity of the courtship conditioning phenotype and to roughly map where EHMT is required to control learning and memory in this paradigm, we performed rescue experiments in the *EHMT^DD2^* background using tissue specific expression of *UAS-EHMT* and short-term memory (30 min after training) as a read-out. The *elav-Gal4* driver was used to express EHMT in all neurons, and the *7B-Gal4* promoter for more selective expression. Indeed, pan-neuronal expression of *EHMT* in the mutant background restored the Learning Index to normal levels (Figure 5e, orange bars, pan neuronal versus EHMT mutants), providing evidence that EHMT is required cell-autonomously in neurons to achieve normal memory. Elav-driven expression of *EHMT* in the *EHMT* ^+^ genetic background had no significant effect on Learning Index (Figure 5e, black bars, pan neuronal versus EHMT mutant). 7B-Gal4 is predominantly expressed in the mushroom bodies of adult brains but is also expressed and at lower levels in some other brain regions, including the antennal lobe (Figure S5) [29]. Expression of EHMT with this driver in the EHMT mutant background was able to rescue the Learning Index (Figure 5e, orange bars, 7B-Gal4 versus EHMT mutant), revealing that EHMT function in 7B-Gal4 neurons is sufficient for normal memory. We also observed that overexpression of *EHMT* using *7B-Gal4* in the *EHMT* ^+^ background significantly reduced the Learning Index (Figure 5e, black bars, 7B-Gal4 versus EHMT mutants). Since the Learning Index was normal in *EHMT* mutants containing both *7B-Gal4* and *UAS-EHMT*, we conclude that there is no deleterious effect due to the expression of *Gal4* or the *7B-Gal4* P-element insertion itself. We therefore asked whether the presence of endogenous EHMT could make a significant difference to the absolute protein levels in the mushroom body upon *7B-Gal4*-mediated overexpression. We observe a very high and uniform EHMT staining in all mushroom body Kenyon cells upon *UAS-EHMT* expression with *7B-Gal4* in the *EHMT^+^* background (Figure S6). A similar staining pattern was observed using this driver in the *EHMT* mutant background, although staining intensity was noticeably lower, likely due to the absence of endogenous EHMT (Figure S6). In contrast, the *elav-Gal4* driver resulted in a non-uniform staining pattern, with high EHMT levels in only a small proportion of Kenyon cells (Figure S6). Thus, overexpression of EHMT in *7B-Gal4* neurons appears to be deleterious when above a certain threshold. These results suggest that appropriate levels of EHMT in the *Drosophila* nervous system are critical for courtship memory and indicate that the requirement for EHMT in this process is confined to *7B-Gal4* positive neurons.

Taken together with the defect in jump reflex habituation, these data reveal an important role for EHMT not only in a simple form of learning but also in a more complex cognitive process such as courtship memory.

### EHMT-Mediated Memory Can Be Rescued in Adulthood

Recently, it has been reported that postnatal loss of Ehmt1 and G9a in mice causes cognitive defects in the absence of obvious developmental abnormalities [15]. We therefore asked whether the memory defects of EHMT mutants in the courtship conditioning paradigm can be rescued by expression of EHMT in adulthood. Indeed, induced expression of EHMT using *hs-Gal4* after eclosion (see Experimental Procedures) completely restored memory defects shown by siblings of the same genotype that had not undergone the heat-shock procedure (Figure 5f). This demonstrates that EHMT is required for memory in adult flies and highlights that cognitive defects are reversible in *EHMT* mutant flies.

### Generation of Genome Wide H3K9me2 Methylation Profiles

**Figure 6:**
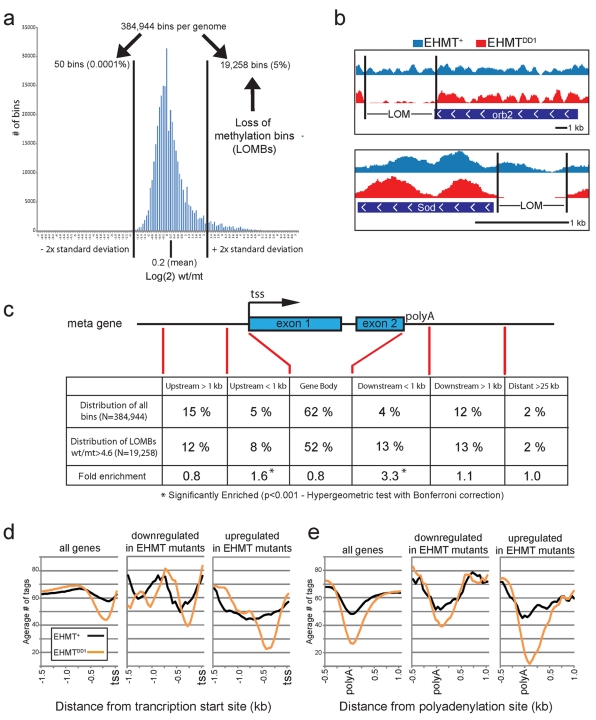
EHMT methylates discrete regions of the euchromatic genome. (a) Histogram showing the distribution of H3K9me2 methylation ratios in logarithmic scale (log # of sequenced tags in wt/# of sequenced tags in mt) after binning of the euchromatic genome into 384,944 300-bp segments. The mean (0.2±1) and 2× the standard deviation is indicated. The region outside +2× the standard deviation is comprised of 19,258 bins, in which methylation is greater than 4.6-fold higher in *EHMT^+^* than in *EHMT^DD1^*. These are referred to as loss of methylation bins (LOMBs). In contrast, the region outside −2× standard deviation contains only 50 bins. (b) Two examples of genomic loci with loss of methylation (LOM) in *EHMT* mutants. The *orb2* genes has a downstream LOM region (top panel) while the *Sod* gene has an upstream LOM region. Scale is indicated. (c) The positional distribution of all bins in relation to genes is compared to the distribution of LOMBs. These distributions are significantly different (*p*<0.001; chi-squared test). LOMBs are significantly enriched within 1 kb upstream of the transcriptional start site (tss) and 1 kb downstream of the polyadenylation site (polyA), by 1.6- and 3.3-fold, respectively (*p*<0.001, hypergeometric test with Bonferroni correction). (d–e) Average number of sequenced tags in *EHMT* ^+^ (black) and *EHMT^DD1^*(orange) upstream of the tss (d) and near the polyA site (e) for all genes (left panels); for genes that are >2.5-fold downregulated in *EHMT* mutants (middle panels); and for genes that are >2.5-fold upregulated in *EHMT* mutants (right panels).

The reversible memory defects in EHMT mutant flies suggest a critical role for EHMT in neuronal function in addition to its role in dendrite development. We therefore wanted to determine the molecular mechanisms through which EHMT regulates neuronal processes. EHMT proteins mediate histone 3 lysine 9 dimethylation (H3K9me2) in euchromatic regions of the mammalian and fly genomes [6],[7],[10]. Therefore, we investigated EHMT target sites by generating genome-wide H3K9me2 profiles for EHMT mutant and wild-type larvae using chromatin immunoprecipitation (ChIP) with an H3K9me2 antibody followed by massive parallel sequencing of the co-precipitated DNA (ChIP-seq technology). Mapping of the sequenced tags to the *Drosophila* genome revealed a genome-wide profile that is consistent with known H3K9me2 patterns [30],[31]. High H3K9me2 is a known characteristic of heterochromatin [31]. Accordingly, we find high H3K9me2 levels in both wild-type and EHMT mutant strains in annotated heterochromatic regions that are contiguous with the assembled euchromatic chromosome arms (Chr2Lh, Chr2Rh, Chr3Lh, Chr3Rh, and ChrXh) (Figure S7) [32]. This was expected, since EHMT is known to have no effect on heterochromatin formation and heterochromatic H3K9me2 levels are known to be unaffected by loss of EHMT/G9a in fly and mouse [7],[15],[19],[33]. The generated H3K9me2 profiles also follow expected patterns in euchromatin. H3K9me2 is known to dip immediately before the transcriptional start site (tss) and near the polyadenylation site (polyA) of genes [30],[34] either due to nucleosome depletion or decreased H3K9me2 in these regions. We indeed observe a dip in H3K9 dimethylation in these regions (Figure 6d and 6e, left panels, black lines), thus demonstrating the accuracy and reliability of our H3K9me2 profiles.

### EHMT Affects H3K9me2 Levels in Discrete Regions of the Euchromatic Genome

Since the global pattern of H3K9me2 appeared to be normal in EHMT mutants, we reasoned that EHMT must affect discrete regions within the genome. To identify these regions we divided the euchromatic genome into 300 bp bins and compared the number of sequenced tags per bin in wild-type versus mutant samples. For each of the 384,944 bins in the euchromatic genome we calculated a methylation ratio by dividing the number of tags in wild-type by the number of tags in the mutant. Thus, a ratio greater than 1 identifies regions where methylation is decreased in *EHMT* mutant flies. We have plotted the log of these ratios (log(2)wt/mt) in a histogram, in which Loss of Methylation Bins (LOMBs) are found in the area of positive log values. The histogram roughly follows a normal distribution but is asymmetric, with 19,258 bins outside two-times the standard deviation of the mean on the positive side, while only 50 bins outside two-times the standard deviation on the negative side (Figure 6a). The 19,258 LOMBs constitute about 5% of the euchromatic genome and provide an unbiased confirmation for the role of EHMT in H3K9 dimethylation. Loss of methylation (LOM) can also be visualized in the USCS genome browser as areas where H3K9me2 levels are depleted in the mutant but remain high in wild-type (two examples given in Figure 6b). Interestingly, we find that LOMBs are not randomly distributed in the genome but are enriched in the areas 1 kb upstream of the tss and 1 kb downstream of the polyA site by 1.6-fold and 3.3-fold, respectively (Figure 6c). As mentioned above, we observe a local depletion of H3K9me2 in these regions in wild-type animals (Figures 6d and 6e, left panel, black line). In EHMT mutants, this local depletion is strongly augmented both upstream of the tss and near the polyA site (Figures 6d and 6e, left panel, orange line), providing further evidence that EHMT deposits H3K9me2 marks in discrete euchromatic loci, with a bias towards the 5′ and 3′ ends of genes.

### Loss of H3K9me2 in EHMT Mutants Can Affect Gene Transcription

H3K9me2 is a marker for condensed, transcriptionally repressive chromatin [35], however the modification itself does not strongly correlate with transcription levels on a genome wide scale as is seen for some other histone modifications, like H3K4me3 and H3K27me3 [30]. To determine whether H3K9me2 can contribute to transcriptional repression in *Drosophila*, we performed microarray expression analysis to compare mRNA levels in *EHMT* wild-type and mutant larvae. We then analyzed H3K9me2 levels upstream of the tss and downstream of the polyA site for genes that were up- and downregulated in EHMT mutants. Genes that are activated by EHMT (i.e. greater that 2.5-fold downregulated in mutants, Table S1) showed no difference in H3K9me2 profiles upstream of the tss or downstream of the polyA site when comparing EHMT wild-type and mutant strains (Figure 6d and 6e, middle panels). In contrast, genes that are repressed by EHMT (i.e. greater that 2.5-fold upregulated in mutants, Table S2) have a clearly augmented dip in methylation both at the tss and polyA sites (Figure 6d and 6e, right panels) when compared to the wild-type profile and to the average methylation profiles of all genes. These data indicate that EHMT-mediated H3K9 dimethylation immediately up and downstream of genes can affect transcriptional repression in *Drosophila.*

### EHMT Target Loci

**Figure 7:**
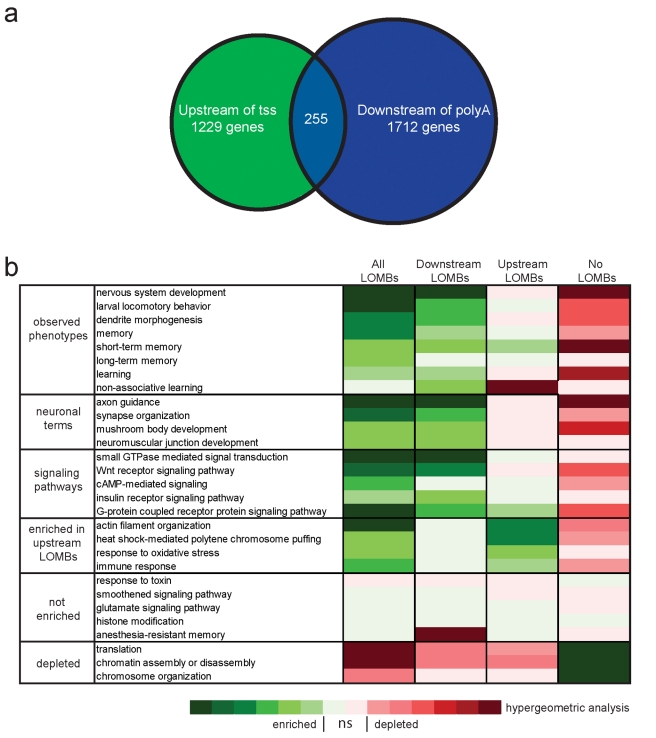
Bioinformatic analysis of EHMT target genes. (a) Venn diagram showing the overlap in genes associated with either Upstream LOMBs or Downstream LOMBs. (b) Heat map showing the significance of enrichment or depletion for gene ontology terms describing biological processes associated with: genes containing LOMBs (All LOMBs—5,136); genes containing LOMBs within 1 kb upstream of the transcriptional start site (Upstream LOMBs—1,229); genes containing LOMBs within 1 kb downstream of the polyA site (Downstream LOMBs—1,712); and genes that do not contain any LOMBs (No LOMBs—10,238). Complete lists of gene IDs for these groups are provided in Table S3. Color scale indicates the significance of enrichment as determined using GOToolBox to perform (http://genome.crg.es/GOToolBox) a hypergeometric test with Benjamini & Hochberg correction. Darkest colors indicate *p* values ≤10^−7^. *ns*  =  non-significant (*p*>0.05).

Next, we investigated which genes were affected by loss of methylation in EHMT mutants by associating each LOMB with its closest gene. In total, LOMBs were found in or near 5,136 genes; 1,229 genes had LOMBs upstream of the tss (upstream LOMB) and 1,712 genes had LOMBs downstream of the polyA site (downstream LOMB) (Table S3). The two groups overlap by 255 genes (Figure 7a).

To assess the function of LOMB-associated genes, we analyzed their gene ontology for enrichment of specific biological processes using GOToolBox [36]. Genes associated with LOMBS are highly enriched in terms related to the nervous system (Figure 7b, for lists of genes associated with selected terms see Table S4). The broad term nervous system development, associated to more than 350 LOMB genes, reaches the highly significant *p* value of 2.3×10^−28^ and is the most enriched tissue-specific term. Consequently, the term is highly depleted from the pool of genes with unaltered H3K9me2 in EHMT mutants; i.e. in genes that are not associated with LOMBs (Figure 7b, no LOMBs). Strikingly, all GO terms that describe *EHMT* mutant phenotypes (e.g. short- and long-term memory, non-associative learning, dendrite morphogenesis, and larval locomotory behavior) show significant enrichment when considering all LOMB-associated genes and genes associated with downstream LOMBs (Figure 7b, observed phenotypes). Other neuronal terms that show high enrichment are also shown (Figure 7b, neuronal terms). Signal transduction is also amongst the most strongly enriched terms, with a *p* value of 6.2×10^−48^. Many specific signaling pathway terms are highly overrepresented amongst LOMB-associated genes, with G-protein coupled receptor protein signaling pathway and small GTPase mediated signal transduction being the top terms (Figure 7b, signaling pathways). We also note significant enrichment of pathway terms that directly relate to EHMT mutant phenotypes, such as cAMP signaling, a major pathway involved in learning and memory.

Notably, there is a stark contrast in enriched terms when comparing genes associated with either upstream or downstream LOMBs (Figure 7b, Downstream LOMBs and Upstream LOMBs). Downstream LOMBs are associated with genes that are enriched for neuronal terms, signaling pathways, and terms describing observed EHMT mutant phenotypes, while upstream LOMBs are associated with genes involved in biological processes requiring a high transcriptional plasticity, such as stress response and actin cytoskeleton remodeling (Figure 7b, enriched in upstream LOMBs). The contrast between these two groups in their gene ontology illustrates the importance of H3K9me2 position at target gene loci and provides further support as to the biological relevance of these data.

Finally, genes involved in regulatory processes such as translation, chromatin assembly/disassembly, and chromosome organization are highly depleted from LOMB-associated genes (Figure 7b, depleted), which contrasts the striking enrichment of nervous system and phenotype-relevant terms amongst LOMB-associated genes.

## Discussion

Here, we demonstrate that *Drosophila* EHMT, a histone methyltransferase, regulates sensory dendrite development, larval locomotory behavior, simple learning (habituation), and complex memory (courtship conditioning). Notably, EHMT mutants are viable, appear healthy, and many other aspects of neuronal development and function are normal, highlighting the selectivity with which EHMT regulates specific aspects of neuronal development and function. Genome-wide molecular analysis of *EHMT* mutant flies supports this idea, revealing altered histone methylation at target loci encompassing a selection of neuronal genes that control learning, memory, and other phenotype-relevant processes.

### EHMT Affects H3K9me2 at Discrete Regions in the Euchromatic Genome

The EHMTs are an evolutionarily conserved family of proteins that regulate H3K9 methylation at euchromatic DNA [6],[7],[10],[15]. Previous studies have shown that EHMTs affect transcription through H3K9 dimethylation in the promoters of certain genes [8],[37]–[39]. Our study provides the first genome-wide overview of EHMT function with respect to its role in post-translational histone modifications. We provide evidence that *Drosophila* EHMT induces H3K9 dimethylation at a proportion (about 5%) of the euchromatic genome, with a preference for discrete regions at the 5′ and 3′ ends of genes (Figure 6). Genes with differential H3K9me2 levels at the 5′ end (within 1 kb upstream of the transcriptional start site) are predominantly involved in biological processes related to stress response (e.g. heat shock response and actin cytoskeleton remodeling), which require rapid and frequent changes in transcription. This observation is consistent with studies in yeast and humans, which show that chromatin structure immediately upstream of transcriptional start sites directly correlates with transcriptional plasticity [40]. In contrast, genes that are differentially methylated at the 3′ end are highly enriched in genes that control neuronal processes that are disrupted in EHMT mutants (Figure 7). The general view is that gene expression is regulated through interactions at the promoter, or 5′ end. However, recent studies have revealed that 3′ gene ends also play an important and complex role in the regulation of transcription by: (1) mediating gene looping [41]–[45], which is necessary for transcriptional memory, i.e. the altered transcriptional responsiveness of genes after a previous cycle of activation and repression [43],[46],[47]; (2) serving as an initiation site for antisense transcripts [41]; and (3) regulating transcript termination, a process that also affects transcript levels [48]. Currently there is no evidence linking H3K9me2 to any of these processes, however it is conceivable that differential histone methylation at the 3′ end of neuronal genes may act as a mechanism to control their expression. In line with this idea a recent study has reported that the DNA methyltransferase, Dnmt3a, also targets neuronal genes in “non-promoter” regions, including 3′ ends [49]. Thus, it appears that epigenetic alterations to non-promoter regions is emerging as a general theme for the regulation of neuronal gene expression.

### Regulation of Dendrite Development and Locomotory Behavior by EHMT

EHMT mutants show a decrease in dendrite branching in sensory neurons of the *Drosophila* peripheral nervous system (Figure 3). Type 4 md neurons are known to provide the sensory input that they receive via their dendrites as an essential functional component to the neuronal circuitry governing larval movement [23]. Our analysis of larval locomotion in *EHMT* mutants revealed a behavioral phenotype characterized by an increased performance of stops, retractions, and turns (Figure 4). It has been reported that such a phenotype can directly arise from dysfunction of type 4 md neurons [22],[24], which raised the possibility that decreased dendrite branching and altered locomotory behavior are connected traits. Re-expression of EHMT in type-4 md neurons did, however, not rescue larval locomotion defects, suggesting that larval locomotion and type 4 md neuron development are controlled independently by EHMT. Thus, this lack of rescue may be due to requirements for EHMT in additional peripheral or central neurons relevant to the crawling pattern. We can also not exclude unspecific secondary effects or that precise levels of re-expressed EHMT may be crucial for turning behavior. Ultimately, the relevance of EHMT in both dendrite development and crawling is illustrated by the observation that EHMT mutants show loss of H3K9me2 at 65 of 147 genes annotated to be involved in dendrite development and 15 of 16 genes involved in larval locomotory behavior (see Table S4 for gene IDs).

### Regulation of Learning and Memory by EHMT

We have shown that EHMT is required for light-off jump reflex habituation (Figure 5a–5c), a simple form of non-associative learning that is known to require classic learning and memory genes such as *rutabaga* [27]. In this paradigm a sequence of leg extension and flight initiation is induced by sudden darkness. This behavioral response is mediated by the giant fiber interneurons, which receive sensory input from the visual system in the brain and relay this information through the thoracic ganglion where efferent neurons descending from the giant fiber to thoracic muscles are stimulated [50],[51]. Only a few genes are known to control jump reflex habituation and most of these are ion channels, or are involved in cAMP and cGMP second messenger signaling pathways [50]. EHMT is the first histone modifying enzyme to be implicated in this simple form of learning. Jump-reflex habituation is not an official gene ontology term, but significantly, seven of the eight genes known to be involved in jump-reflex habituation [50] show loss of H3K9 dimethylation in EHMT mutants (Table S4).

We have also identified a role for *EHMT* in courtship memory (Figure 5d–5f). This is a complex form of memory that allows male flies to discriminate between receptive and non-receptive females, presumably to optimize the energy that they spend on courtship. We demonstrate that loss of *EHMT* leads to impaired short- and long-term memory while the learning capacity of the EHMT mutants was unaffected (Figure 5d). Moreover, we show that normal courtship memory is restored upon re-expression of EHMT in the whole nervous system and in a subset of neurons labeled by *7B-Gal4*, which is predominantly expressed in the mushroom body neurons of the adult brain (Figure 5e). Although further work is required to map the specific circuits required for EHMT-dependent courtship memory, the mushroom body is known to be crucial for courtship memory, but not learning [52], pointing towards a deficit in this area of the brain. Significantly, EHMT affects histone methylation in 22 of 36 genes that were annotated at the time of our analysis to be involved in memory (Table S4). Other relevant memory genes, such as *Orb2* [53] (Figure 6b), *nemy* [54], and *ben* [55], that were not yet included in gene ontology databases are also affected by loss of *EHMT*. Together, these data suggest that EHMT targets two-thirds of all currently known memory genes.

Importantly, we were able to fully restore memory deficits by re-expression of EHMT during adulthood (Figure 5f). Thus, although EHMT can affect neuronal hardwiring (dendrite development in the peripheral nervous system; Figure 3), it appears that adult cognitive defects do not arise from neurodevelopmental defects occurring prior to eclosion. This is consistent with a recently reported impairment in fear conditioning that has been observed in mice with postnatal loss of Ehmt1 in the brain [15] and with our observation that mushroom body morphology appears unaffected in EHMT mutant flies. Thus, EHMT-mediated H3K9 dimethylation of specific loci is required in adult post-mitotic neurons to consolidate or retrieve consolidated memories. Interestingly, other epigenetic regulators, such as the DNA methyltransferases Dnmt1 and Dnmt3a, are also required in post-mitotic neurons for normal memory [56]. These studies support the idea that the process of learning induces reprogramming of the neuronal epigenome, which crucially underlies memory [1]–[3]. Such “stable” chromatin modifications, including DNA and histone methylation, appear to be good candidates for “writing” long-term memory, however these marks must also remain dynamic allowing for memories to be modified. Our understanding of this stable versus dynamic state of epigenetics in neurons and its consequences are highly limited. It will thus be important to dissect the extent of epigenetic plasticity during the different phases of learning, memory consolidation, and memory retrieval, and to determine how these alterations to the epigenetic landscape translate into transcriptional changes required for information processing and storage.

A recent study of mRNA levels in mice with brain region-specific loss of Ehmt1 has identified 56 genes that are consistently misregulated in the mutant mouse brain [15]. Of these 56 genes, 18 are non-neuronal, which led to the interpretation that EHMT proteins control cognition through repression of non-neuronal genes in neuronal tissues. In contrast to this view, our data show that *Drosophila* EHMT mediates H3K9 dimethylation at more than 350 neuronal gene loci with proven critical roles in nervous system development and function. Does this apparent discrepancy reflect evolutionary differences? Of the 56 differentially expressed genes identified by Schaefer et al. [15], 30 are conserved in flies and 20 show loss of H3K9me2 in EHMT mutants (Table S4). This correlation is very unlikely to occur by chance (*p*<2.9×10^−4^; hypergeometric test), suggesting that EHMT target genes are, at least in part, evolutionarily conserved. The great number of highly enriched neuronal genes amongst *Drosophila* EHMT targets, their striking match with EHMT mutant phenotypes, and the reversibility of cognitive defects argue that EHMT orchestrates an epigenetic program that directly regulates a battery of neuronal players underlying the molecular basis of cognition. It is also noteworthy that EHMT targets include fly orthologs of *NF1*, *FMR1*, *FMR2*, *CNTNAP2*, *GDI*, *DLG3*, and of many more genes underlying syndromic and non-syndromic forms of intellectual disability. Also, the major signaling pathways known to underlie intellectual disability, Rho and Ras GTPase pathways [57],[58], are highly enriched in our ontology analysis (GO term: small GTPase mediated signal transduction).

Our study complements a number of reports on post-embryonic rescue of cognitive phenotypes in disease models of intensively studied disorders such as Fragile X syndrome, Neurofibromatosis I, Tuberous sclerosis, Rubinstein-Taybi, Angelman, and Rett syndrome [59]. The growing number of such examples provides an argument for reappraisal of the traditional view that genetic forms of intellectual disability are largely due to irreversible neurodevelopmental defects, findings which open prospects for therapeutic intervention. Currently, clinical trials are underway to treat Fragile X patients with compounds that have initially been identified to rescue phenotypes in fly models of Fragile X syndrome [60]–[63]. The EHMT mutant fly has provided novel insights into the epigenetic regulation of cognition and will be a valuable tool to work further towards such translational approaches. Furthermore, a better understanding of the epigenetic mechanisms regulating cognitive processes is relevant to the wider medical community, considering the increased awareness of the epigenetic contributions to neurodevelopmental and psychiatric disorders in general [4],[5].

## Materials and Methods

### Fly Stocks and Maintenance

Flies were reared on standard medium (cornmeal/sugar/yeast) at 25 degrees and 45%–60% humidity with a 12-h light/dark cycle. All fly stocks were obtained from the Bloomington *Drosophila* stock center (Indiana University) (see Text S1 for stock descriptions) except for EHMT deletion strains and UAS-EHMT strains, which were generated according to standard procedures (see Text S1 and Figure 2).

### Immunohistochemisty and Stainings

Tissues were dissected and fixed using standard methods. Rabbit-anti-EHMT antibodies were a gift from Dr. A. Lambertson [10] and were used at a 1/100. Rat-anti-elav (1/500), mouse-anti-repo (1/500), mouse-anti-DLG (1/100), and mouse anti-dac (1/100) antibodies were obtained from the Developmental Studies Hybridoma Bank (University of Iowa). Nuclei were visualized using the fluorescent nuclear dye DAPI. For imaging of type IV md neurons expressing a membrane targeted mCD8-GFP fusion protein (memGFP) we used a rat-anti-mCD8 antibody (Invitrogen) at 1/100. Secondary antibodies were conjugated to either alexa-fluor-568 or alexa-fluor-488 (Invitrogen). Images were acquired using either a Leica DM-IRE2 confocal microscope (Leica Microsystems) or a Zeiss Axioimager Z1 fluorescent microscope equipped with an ApoTome (Carl Zeiss B.V.). Where possible, colocalization was shown in a color-blind-friendly manner using photoshop to copy red or blue signals into both the red and blue channel to produce magenta.

### Western Blotting

Proteins were extracted from 0–3 h embryo collections as previously described [64] and subjected to Western blot analysis according to standard procedures using the Bio-Rad electrophoresis system (Bio-Rad) (see Text S1 for details).

### Analysis of md Neurons

We have analyzed the morphology of the solitary type 4 md neuron in the ventral cluster called vdaB (ventral dendritic arborization neuron B) [18]. For the visualization of dendritic arbors we used the type 4 md neuron-specific driver *477-Gal4* [20] to drive expression of memGFP. Details of crosses, confocal microscopy, and quantification of dendrite ends are provided in Text S1.

### Analysis of Larval Locomotory Behavior

Larval crawling was assayed as described previously (refer to Text S1 for details) [25]. Approximately 30 individuals per strain were tested per day over a 5-d period, resulting in a total of approximately 150 larvae per genotype and experiment. Experiments were performed at least twice. Quantification of path lengths was performed using Adobe Photoshop and Image J.

### Light-Off Jump Reflex Habituation

Male flies of the genotypes *EHMT^+^/Y*, *EHMT^DD1^/Y*, *EHMT^DD2^/Y*, and female flies of the genotypes *EHMT^+^/EHMT^+^* and *EHMT^DD1^/EHMT^DD2^* were tested for light-off jump reflex habituation in a modified assay that was previously described by Engel and Wu [27]. Details of this high throughput assay are described Text S1.

### Courtship Conditioning

Flies were tested for learning and/or memory at 4 d of age using the courtship conditioning assay as previously described [53]. For induced EHMT expression via *hs-Gal4*, flies were incubated at 37 degrees for 45 min on days 1–3, with the final heat shock treatment taking place 24 h before training and testing on day 4.

### ChIP-seq

Chromatin immunoprecipitation was performed using standard methods with anti-H3K9me2 antibodies (07-441, Upstate) and Prot A/G beads (Santa Cruz) to capture antibody bound chromatin (for details see Text S1). Massive-parallel sequencing was performed using the Illumina Genome Analyzer IIx according to standard protocols of the manufacturer (Illumina) (for details see Text S1). All sequence analyses were conducted using the BDGP Release 5 genome assembly (DM3) and the release 5.12 annotations provided by FlyBase. To compensate for differences in sequencing depth and mapping efficiency among the two ChIP-seq samples, the total number of unique tags of each sample was uniformly equalized relative to the sample with the lowest number of tags (7,043,913 tags), allowing for quantitative comparisons. For association of individual bins with genes, we determined the distances from the middle of the bin to the nearest tss or polyA site using the Pinkthing tool (http://pinkthing.cmbi.ru.nl/cgi-bin/index50.pl). The ChIP-Seq data from this study are available at the NCBI Gene Expression Omnibus (http://www.ncbi.nlm.nih.gov/geo/) under series accession no. GSE22447.

### Mircroarray Expression Analysis

Total RNA was isolated in triplicate from third instar larvae using the RNeasy Lipid Tissue Midi kit (Qiagen). RNA quality was evaluated using spectrophotometry and integrity was confirmed using gel electrophoresis of glyoxal-denatured samples. Total RNA samples were labeled using the ‘indirect’ method [65]. Superscript II reverse transcriptase (Invitrogen) was used to produce cDNA incorporated with aminoallyl-dUTP (Fermentas). Reactive fluorescent dyes (Alexa647 or Alexa555; Invitrogen) were conjugated to the individual samples. Two differently labeled samples, whole larvae from *EHMT* mutant versus *EMHT^+^* wild-type, were pooled and co-hybridized to the 14K long oligo array from the Canadian Drosophila Microarray Centre (www.flyarrays.com) according to previously described methods [66]. Images of the hybridized microarrays were obtained using a ScanArray 4000 scanner (Perkin-Elmer) and were quantified using QuantArray 3.0 software (Perkin-Elmer). Data were normalized using lowess sub-grid normalization using Genetraffic Duo (Stratagene) analysis software. Normalized data were exported and analyzed using the one-class test available in the Statistical Analysis of Microarrays (SAM) software package. The false discovery rate of the one-class test was adjusted such that the expected number of false positive results was less than one. Gene lists generated in SAM were filtered to include only those genes that displayed at least a 2.5-fold increase or decrease in abundance with respect to the wild-type sample and whose coefficient of variance was less than 100%.

### Statistical Analysis

For all data, normal versus non-normal distribution was assessed using the Shapiro-Wilk test and by visual examination of histograms. For comparison of more than two variants with a normal distribution, one-way ANOVA analysis was used to determine the probability that there were differences between the variants. In the cases that ANOVA indicated that there was a significant differences between variants (*p*<0.05) we performed post hoc pair-wise comparisons using the Bonferroni correction, which takes into account that multiple comparisons are being made and therefore increases the stringency of the test. This method was applied for normally distributed data in Figures 3c, 5c, S2e, S2f, S3, and S4b. For comparison of more than two variants with a non-normal distribution, the Kruskal-Wallis test was used to determine if there were significant differences between any of the means. For data sets in which there was a significant difference (*p*<0.05), we subsequently performed pair-wise comparisons using the Mann-Whitney test, a post hoc test that can be used to compare two means with non-normal distributions. The combination of Kruskal-Wallice and Mann-Whitney was used for data sets which were not normally distributed (Figures 4b–4c and 5d–5f). All of the above tests were performed using SPSS software (SPSS Inc.).

To test whether the number of LOMBs in a given genomic position (e.g. gene body) was significantly enriched compared to the distribution of all bins in the genome, we applied a hypergeometric test using an online tool (http://stattrek.com/Tables/Hypergeometric.aspx). This test was performed for all genomic regions defined in Figure 6c—Upstream >1 kb, Upstream <1 kb, Gene Body, Downstream <1 kb, Downstream >1 kb, and Distant—to obtain individual *p* values. Since we performed this test for six genomic regions, *p* values were corrected using the Bonferroni method to account for multiple comparisons using the same data set.

Enrichment of gene ontolology terms was analyzed using GOToolBox [36] to perform a hypergeometric test with Benjamini & Hochberg correction.

## Supporting Information

Figure S1 **Localization of EHMT in type 4 md neurons, adult brains, and the larval body wall.** (a) A type 4 multiple dendrite neuron, vdaB, of the larval skin labeled with memGFP (green) using the *477-Gal4* driver and stained with anti-EHMT (red) and DAPI (blue). Shown here is a representative image from *EHMT^DD1^*. (b) Confocal sections of adult brains stained with anti-EHMT (green) and anti-dac (magenta). Dac is present in the nuclei of the mushroom body Kenyon cells. EHMT labels dac positive cells in wild-type (top) but is absent in EHMT mutants (bottom). (c) Larval body wall stained with anti-EHMT (green) and DAPI (magenta). The larval body wall primarily consists of muscle and epidermal cells; some examples of these cell types are labeled with arrows and arrow heads, respectively. EHMT appears to be present in all nuclei in wild type but is absent in *EHMT^DD1^*.
(2.42 MB TIF)

Figure S2 **mRNA levels of** ***CG3038*** **.** Quantitative Real time qPCR was used to measure the relative levels of *CG3038* mRNA in *EHMT^DD1^* mutant larvae and *EHMT* ^+^ larvae. Data shown are the average relative expression obtained using three reference genes, *β'cop*, *eIF2b-γ*, and *RpII140*.
(0.14 MB TIF)

Figure S3 **EHMT does not affect adult mushroom body morphology, larval neuromuscular junction morphology, or adult photoreceptor function.** (a–b) Adult mushroom bodies from (a) *EHMT^+^* and (b) *EHMT^DD2^* were visualized by expression of *UAS-memGFP* with *7B-Gal4*. (c–d) Larval muscle 4 neuromuscular junctions from (c) *EHMT^+^* and (d) *EHMT^DD2^* were visualized by anti-DLG labeling. (e) Quantification of NMJ area revealed no difference between *EHMT* ^+^, *EHMT^DD1^*, and *EHMT^DD2^*. (f) Quantification of bouton number revealed a slight decrease of about 4 boutons in *EHMT^DD2^*, but no difference between *EHMT* ^+^ and *EHMT^DD1^*. For quantification of NMJ area and button number *n* = 52, 53, and 59 for *EHMT* ^+^, *EHMT^DD1^*, and *EHMT^DD2^*, respectively. (g) Electroretinograms from *EHMT* mutant and wild type adults show that *EHMT* mutant flies have normal photoreceptor function. Error bars represent standard error of the mean.
(0.70 MB TIF)

Figure S4 **Loss of EHMT does not affect phototaxis or negative geotaxis.** Mean (a) phototaxis index and (b) climbing index in *EHMT^+^*, *EHMT^DD1^*, and *EHMT^DD2^*. No significant differences were found between the three genotypes. Error bars represent standard error of the mean.
(0.19 MB TIF)

Figure S5 **Expression of 7B-Gal4 in the adult brain.** Confocal projections of the anterior (top) and posterior (bottom) regions of the adult brain in *UAS-GFP/+; 7B-Gal4/+* flies. GFP expression is observed predominantly in the mushroom body (mb) and mushroom body calyx (mbc). Lower levels of staining are seen in the antennal lobe (AL), lobula (lo), and what appears to be the suboesophogeal ganglion (sog).
(0.82 MB TIF)

Figure S6 **EHMT protein levels in the mushroom body upon expression with** ***7B-Gal4*** **and** ***elav-Gal4.*** (A) Whole mount adult brains were stained with anti-EHMT antibodies and anti-dac antibodies, which label the nuclei of mushroom body cells (Kenyon cells). Using *7B-Gal4* (left panels) EHMT protein is observed at a high level in all dac positive cells. Absolute protein levels are lower in the *EHMT^DD1^* background, likely due to absence of the endogenous protein. Using elav-Gal4, EHMT expression also appears at a high level in Kenyon cells, however only in a subset of these cells. (B) Image J was used to quantify EHMT levels. We measured EHMT staining intensity in dac positive regions of the brain. Overall fluorescence is highest in *EHMT^+^; 7BGal4/UAS-EHMT*, correlating with the loss of learning in this genetic condition. The other three genotypes show a significantly lower overall staining level (*p*<0.01). Error bars represent standard error of the mean.
(1.81 MB TIF)

Figure S7 **Chromosome-wide H3K9 dimethylation in** ***EHMT*** **wild-type and mutant strains.** Sequenced tags, isolated by ChIP with H3K9me2 antibodies, were mapped to the *Drosophila* genome and visualized using the USCS genome browser. All chromosomes show an increase in H3K9me2 at the centromeric end of the chromosomes (labeled 2Lh, 2Rh, 3Lh, and 3Rh and marked with black bars). This is expected since these regions are known to have heterochromatic properties and are contiguous with centromeric heterochromatin. The increase is less pronounced in Chromosome 3Rh, which is known to be more similar to euchromatin than the other centromeric ends, 3Lh, 2Lh, and 2Rh.
(0.44 MB TIF)

Table S1 **Genes downregulated 2.5-fold or more in** ***EHMT*** **mutant larvae as compared to** ***EHMT^+^.***
(0.04 MB DOC)

Table S2 **Genes upregulated 2.5-fold or more in** ***EHMT*** **mutant larvae as compared to** ***EHMT^+^.***
(0.05 MB DOC)

Table S3 **LOMB-associated genes.**
(1.11 MB XLS)

Table S4 **Genes associated with official gene ontology terms or otherwise annotated gene groups.** ^a^Official ontology term according to www.geneontology.org. ^b^From Engel and Wu, 2009. ^c^Mammalian-to-fly orthology refers to one-to-one or many-to-one orthology. ^d^Schaefer et al., 2009 [15]. ^e^Fly genes represented by more than one mouse counterpart in Schaefer et al., 2009 [15].
(0.03 MB DOC)

Text S1 **Supplementary Materials, Methods, and Results.**
(0.09 MB DOC)

## Abbreviations

CI: Courtship Index
DD: downstream deletion
EHMT: euchromatin histone methyltransferase
H3K9: histone 3 at lysine 9
LI: Learning Index
LOMB: loss of methylation bin
LTM: long term memory
STM: short term memory
