# Supplementary figures and images for: Epigenetic Regulation of Learning and Memory by Drosophila EHMT/G9a
Source: PLoS Biol. 2011 Jan 4;9(1):e1000569. doi: 10.1371/journal.pbio.1000569 (PMC3014924; doi:10.1371/journal.pbio.1000569)

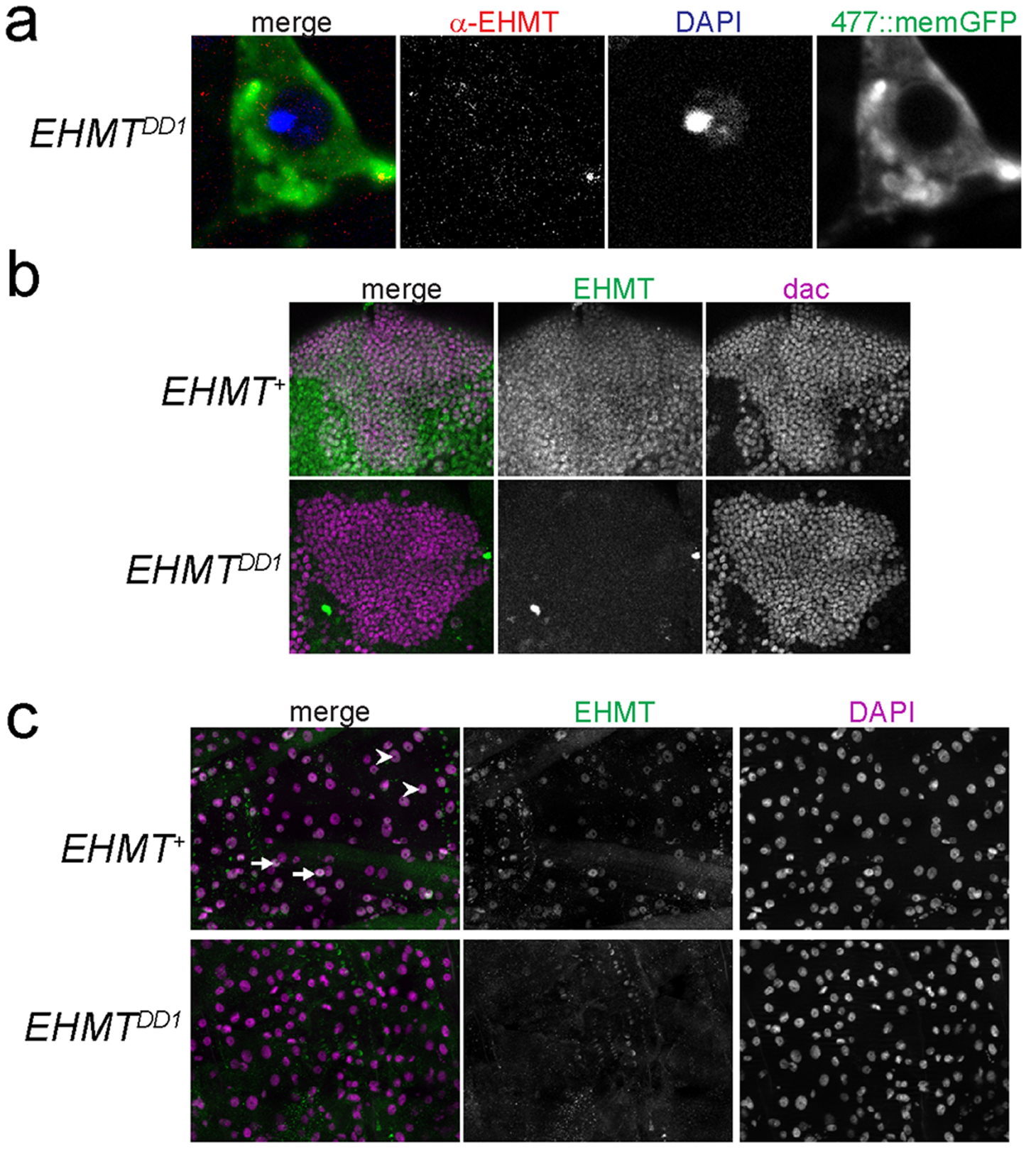

Supplement: Figure S1 — Localization of EHMT in type 4 md neurons, adult brains, and the larval body wall. (a) A type 4 multiple dendrite neuron, vdaB, of the larval skin labeled with memGFP (green) using the 477-Gal4 driver and stained with anti-EHMT (red) and DAPI (blue). Shown here is a representative image from EHMTDD1. (b) Confocal sections of adult brains stained with anti-EHMT (green) and anti-dac (magenta). Dac is present in the nuclei of the mushroom body Kenyon cells. EHMT labels dac positive cells in wild-type (top) but is absent in EHMT mutants (bottom). (c) Larval body wall stained with anti-EHMT (green) and DAPI (magenta). The larval body wall primarily consists of muscle and epidermal cells; some examples of these cell types are labeled with arrows and arrow heads, respectively. EHMT appears to be present in all nuclei in wild type but is absent in EHMTDD1. (2.42 MB TIF) [file pbio.1000569.s001.tif]

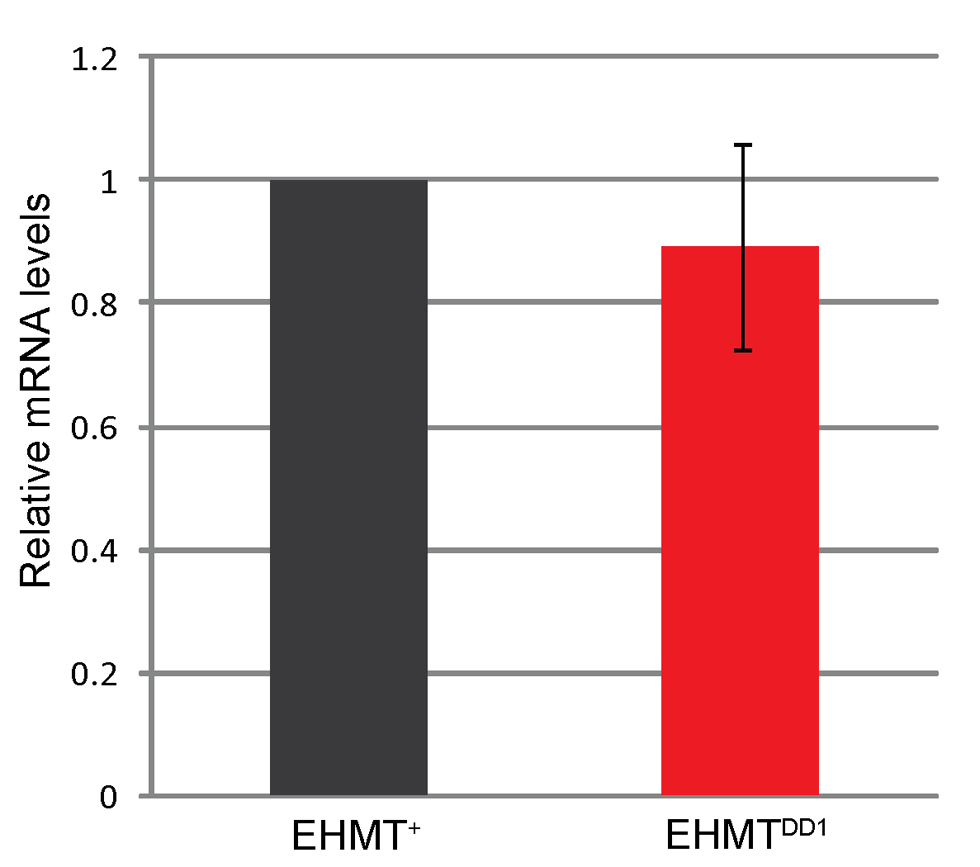

Supplement: Figure S2 — mRNA levels of CG3038 . Quantitative Real time qPCR was used to measure the relative levels of CG3038 mRNA in EHMTDD1 mutant larvae and EHMT + larvae. Data shown are the average relative expression obtained using three reference genes, β'cop, eIF2b-γ, and RpII140. (0.14 MB TIF) [file pbio.1000569.s002.tif]

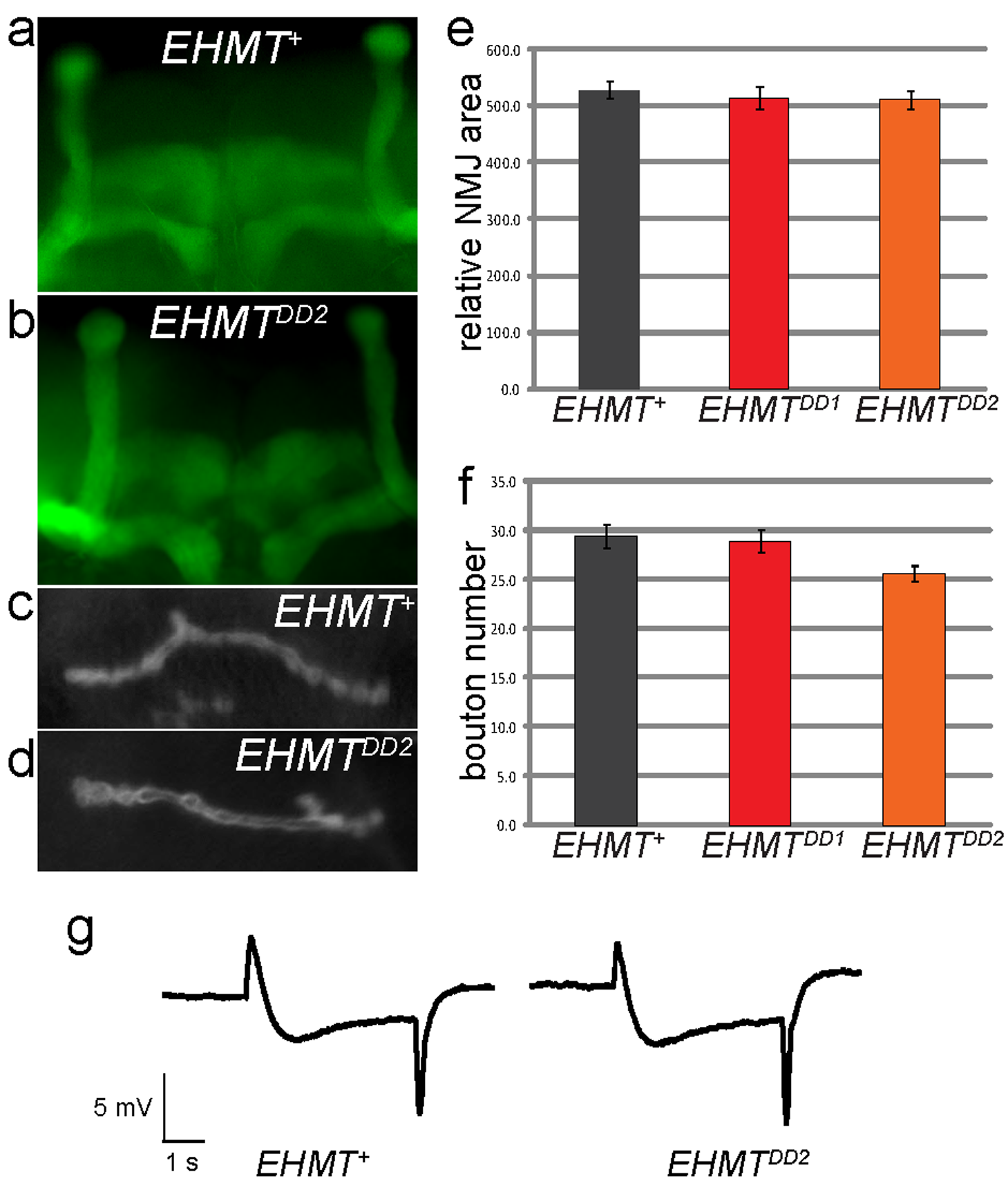

Supplement: Figure S3 — EHMT does not affect adult mushroom body morphology, larval neuromuscular junction morphology, or adult photoreceptor function. (a–b) Adult mushroom bodies from (a) EHMT+ and (b) EHMTDD2 were visualized by expression of UAS-memGFP with 7B-Gal4. (c–d) Larval muscle 4 neuromuscular junctions from (c) EHMT+ and (d) EHMTDD2 were visualized by anti-DLG labeling. (e) Quantification of NMJ area revealed no difference between EHMT +, EHMTDD1, and EHMTDD2. (f) Quantification of bouton number revealed a slight decrease of about 4 boutons in EHMTDD2, but no difference between EHMT + and EHMTDD1. For quantification of NMJ area and button number n = 52, 53, and 59 for EHMT +, EHMTDD1, and EHMTDD2, respectively. (g) Electroretinograms from EHMT mutant and wild type adults show that EHMT mutant flies have normal photoreceptor function. Error bars represent standard error of the mean. (0.70 MB TIF) [file pbio.1000569.s003.tif]

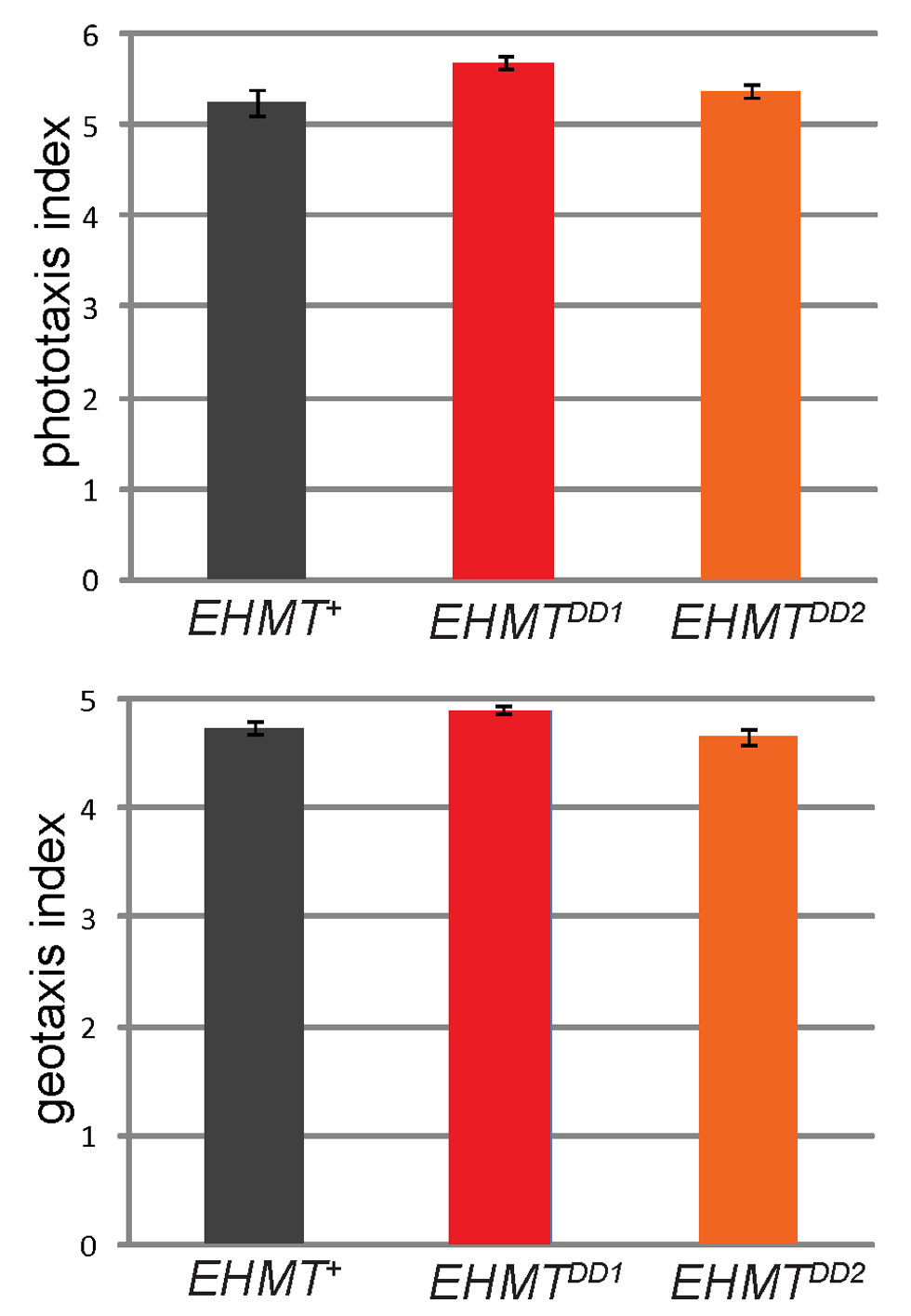

Supplement: Figure S4 — Loss of EHMT does not affect phototaxis or negative geotaxis. Mean (a) phototaxis index and (b) climbing index in EHMT+, EHMTDD1, and EHMTDD2. No significant differences were found between the three genotypes. Error bars represent standard error of the mean. (0.19 MB TIF) [file pbio.1000569.s004.tif]

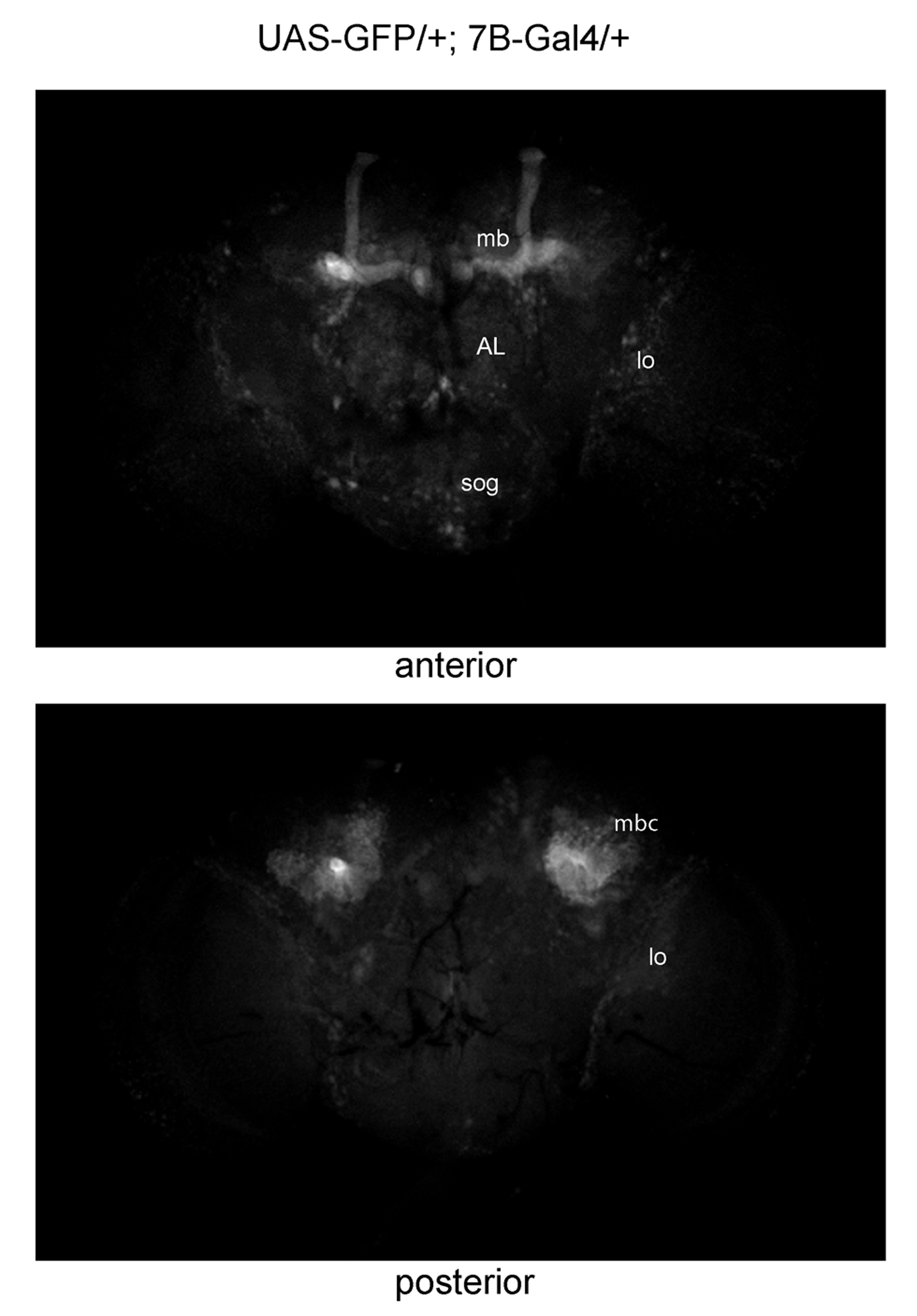

Supplement: Figure S5 — Expression of 7B-Gal4 in the adult brain. Confocal projections of the anterior (top) and posterior (bottom) regions of the adult brain in UAS-GFP/+; 7B-Gal4/+ flies. GFP expression is observed predominantly in the mushroom body (mb) and mushroom body calyx (mbc). Lower levels of staining are seen in the antennal lobe (AL), lobula (lo), and what appears to be the suboesophogeal ganglion (sog). (0.82 MB TIF) [file pbio.1000569.s005.tif]

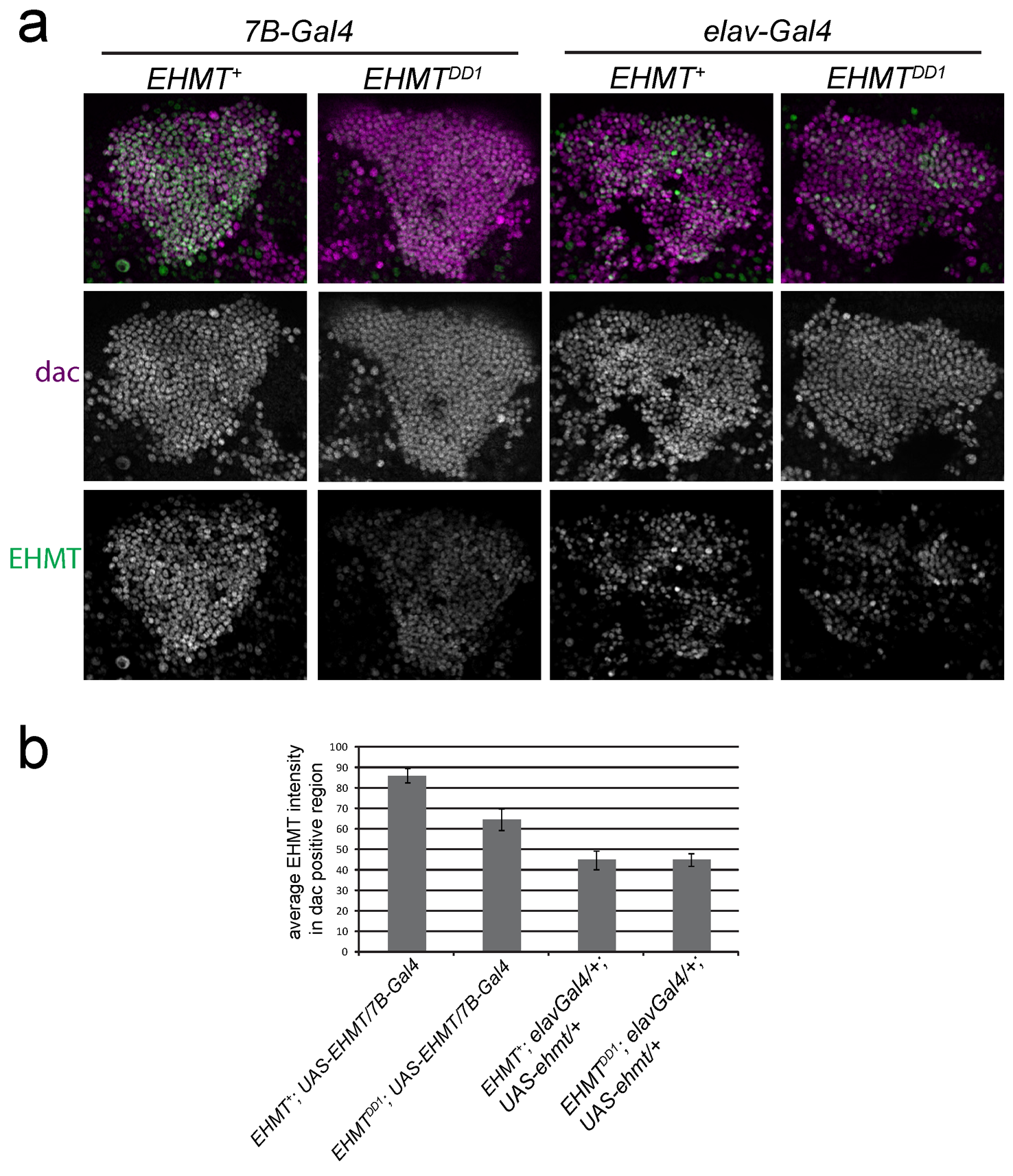

Supplement: Figure S6 — EHMT protein levels in the mushroom body upon expression with 7B-Gal4 and elav-Gal4. (A) Whole mount adult brains were stained with anti-EHMT antibodies and anti-dac antibodies, which label the nuclei of mushroom body cells (Kenyon cells). Using 7B-Gal4 (left panels) EHMT protein is observed at a high level in all dac positive cells. Absolute protein levels are lower in the EHMTDD1 background, likely due to absence of the endogenous protein. Using elav-Gal4, EHMT expression also appears at a high level in Kenyon cells, however only in a subset of these cells. (B) Image J was used to quantify EHMT levels. We measured EHMT staining intensity in dac positive regions of the brain. Overall fluorescence is highest in EHMT+; 7BGal4/UAS-EHMT, correlating with the loss of learning in this genetic condition. The other three genotypes show a significantly lower overall staining level (p<0.01). Error bars represent standard error of the mean. (1.81 MB TIF) [file pbio.1000569.s006.tif]

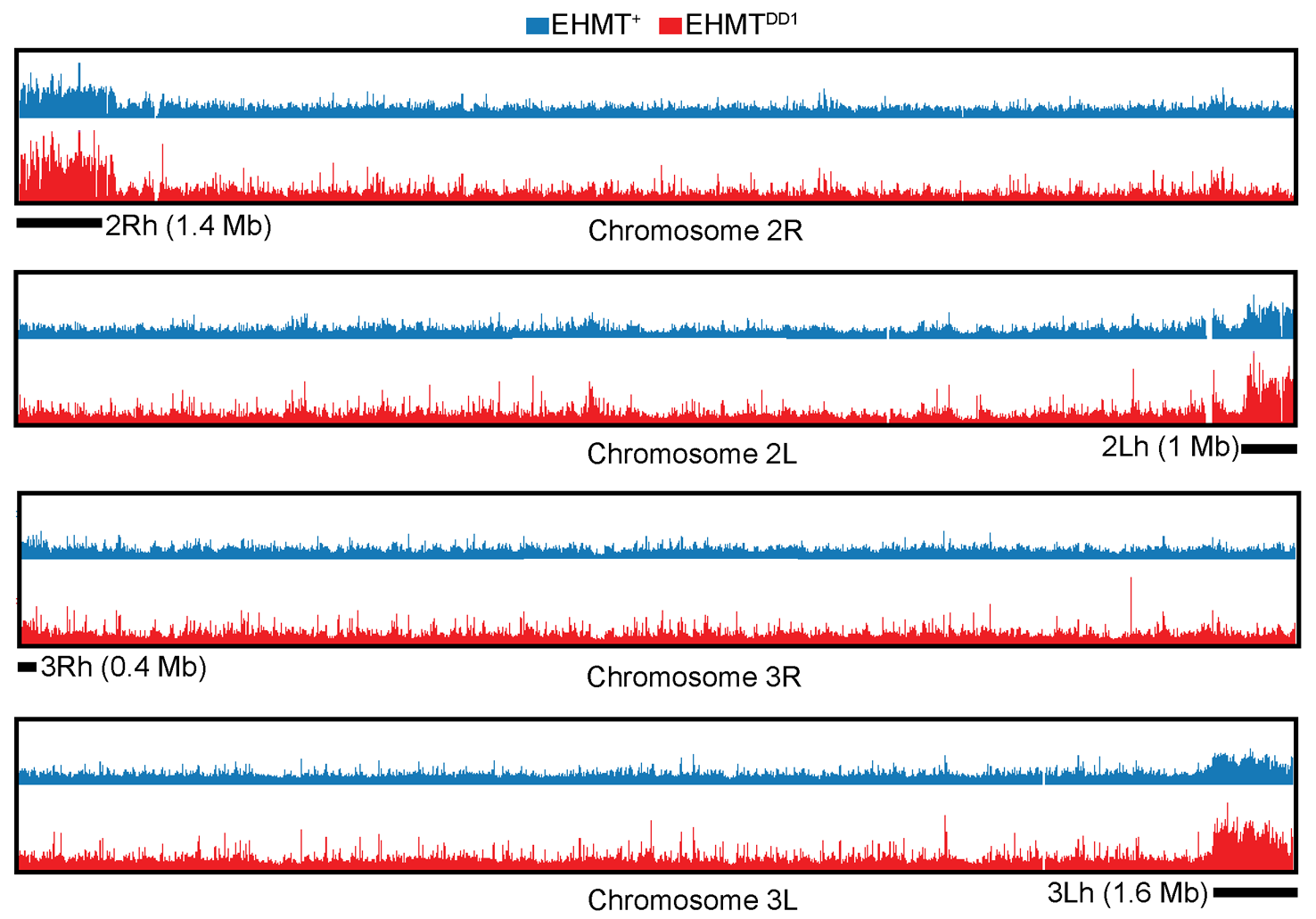

Supplement: Figure S7 — Chromosome-wide H3K9 dimethylation in EHMT wild-type and mutant strains. Sequenced tags, isolated by ChIP with H3K9me2 antibodies, were mapped to the Drosophila genome and visualized using the USCS genome browser. All chromosomes show an increase in H3K9me2 at the centromeric end of the chromosomes (labeled 2Lh, 2Rh, 3Lh, and 3Rh and marked with black bars). This is expected since these regions are known to have heterochromatic properties and are contiguous with centromeric heterochromatin. The increase is less pronounced in Chromosome 3Rh, which is known to be more similar to euchromatin than the other centromeric ends, 3Lh, 2Lh, and 2Rh. (0.44 MB TIF) [file pbio.1000569.s007.tif]
