# Supplementary material for: Epigenetic Regulation of Learning and Memory by Drosophila EHMT/G9a
Source: PLoS Biol. 2011 Jan 4;9(1):e1000569. doi: 10.1371/journal.pbio.1000569 (PMC3014924; doi:10.1371/journal.pbio.1000569)
Supplement: Table S1 — Genes downregulated 2.5-fold or more in EHMT mutant larvae as compared to EHMT+. (0.04 MB DOC) [file pbio.1000569.s008.doc]

**Table S1: Genes downregulated 2.5 fold or more in *EHMT* mutant larvae as compared to *EHMT^+^*.**

| **Gene Symbol** | **Flybase ID** | **Mean Log(2) ratio** | **Fold Change in Expression** |
| --- | --- | --- | --- |
| CG13992 | FBgn0031756 | -1.34 | -2.53 |
| CG8713 | FBgn0033257 | -1.34 | -2.53 |
| LysS | FBgn0004430 | -1.38 | -2.60 |
| LysC | FBgn0004426 | -1.39 | -2.62 |
| Muc68E | FBgn0053265 | -1.48 | -2.78 |
| htt | FBgn0027655 | -1.49 | -2.80 |
| MtnC | FBgn0038790 | -1.53 | -2.88 |
| l(2)01810 | FBgn0010497 | -1.55 | -2.92 |
| LysB | FBgn0004425 | -1.61 | -3.05 |
| CG1702 | FBgn0031117 | -1.65 | -3.13 |
| CG14151 | FBgn0036089 | -1.66 | -3.16 |
| CG14694 | FBgn0037845 | -1.75 | -3.36 |
| CG17134 | FBgn0032304 | -1.75 | -3.36 |
| CG18404 | FBgn0039761 | -1.78 | -3.43 |
| CG5470 | FBgn0038384 | -2.66 | -6.32 |
| CG10361 | FBgn0036208 | -3.44 | -10.85 |
