# Supplementary material for: Epigenetic Regulation of Learning and Memory by Drosophila EHMT/G9a
Source: PLoS Biol. 2011 Jan 4;9(1):e1000569. doi: 10.1371/journal.pbio.1000569 (PMC3014924; doi:10.1371/journal.pbio.1000569)
Supplement: Table S2 — Genes upregulated 2.5-fold or more in EHMT mutant larvae as compared to EHMT+. (0.05 MB DOC) [file pbio.1000569.s009.doc]

**Table S2: Genes upregulated 2.5 fold or more in *EHMT* mutant larvae as compared to *EHMT^+^*.**

| **Gene ID** | **Flybase ID** | **Mean Log(2) ratio** | **Fold Change in Expression** |
| --- | --- | --- | --- |
| CG4467 | FBgn0039064 | 1.34 | +2.53 |
| CG34437 | FBgn0085466 | 1.35 | +2.54 |
| CG30267 | FBgn0050267 | 1.35 | +2.54 |
| CG5590 | FBgn0039537 | 1.37 | +2.58 |
| CG18107 | FBgn0034330 | 1.38 | +2.60 |
| CG15404 | FBgn0031512 | 1.39 | +2.62 |
| dro6 | FBgn0052268 | 1.4 | +2.63 |
| proPO-A1 | FBgn0261362 | 1.4 | +2.63 |
| CG4302 | FBgn0027073 | 1.42 | +2.67 |
| ATPsyn-gamma | FBgn0020235 | 1.43 | +2.69 |
| CG17325 | FBgn0040993 | 1.53 | +2.88 |
| CG8664 | FBgn0030836 | 1.53 | +2.88 |
| IM3 | FBgn0040736 | 1.57 | +2.96 |
| IM2 | FBgn0025583 | 1.64 | +3.11 |
| CG4229 | FBgn0036639 | 1.65 | +3.13 |
| CG31698 | FBgn0051698 | 1.68 | +3.20 |
| CG16836 | FBgn0040735 | 1.7 | +3.24 |
| CG42750 | FBgn0259819 | 1.81 | +3.50 |
| CG6283 | FBgn0039474 | 1.84 | +3.58 |
| CG4115 | FBgn0038017 | 1.88 | +3.68 |
| CG6640 | FBgn0036068 | 1.91 | +3.75 |
| CG3264 | FBgn0034712 | 1.95 | +3.86 |
| Jon74E | FBgn0023197 | 2.18 | +4.53 |
| IM1 | FBgn0034329 | 2.21 | +4.62 |
| CG15068 | FBgn0040733 | 2.24 | +4.72 |
| CG6277 | FBgn0039475 | 2.3 | +4.92 |
| CG3292 | FBgn0034710 | 2.46 | +5.50 |
| CG7587 | FBgn0038523 | 2.53 | +5.77 |
| Amy-d | FBgn0000078 | 2.54 | +5.81 |
| CG17105 | FBgn0032280 | 3.3 | +9.84 |
