# Supplementary material for: Epigenetic Regulation of Learning and Memory by Drosophila EHMT/G9a
Source: PLoS Biol. 2011 Jan 4;9(1):e1000569. doi: 10.1371/journal.pbio.1000569 (PMC3014924; doi:10.1371/journal.pbio.1000569)
Supplement: Table S4 — Genes associated with official gene ontology terms or otherwise annotated gene groups. aOfficial ontology term according to www.geneontology.org. bFrom Engel and Wu, 2009. cMammalian-to-fly orthology refers to one-to-one or many-to-one orthology. dSchaefer et al., 2009 [15]. eFly genes represented by more than one mouse counterpart in Schaefer et al., 2009 [15]. (0.03 MB DOC) [file pbio.1000569.s011.doc]

**Table S4: Genes associated with official gene ontology terms or otherwise annotated gene groups.**

| **GO term or otherwise annotated group** | **LOMB-associated genes** |
| --- | --- |
| Dendrite morphogenesis^a^ | *sv, Smox, ham, EcR, dally, chinmo, CG34340, Fmr1, CG2678, bon, scrt, jumu, nerfin-1, d4, wit, MEP-1 ,Mi-2, Elongin-C, lbe, Sema-1a, run, vvl, Lis1, comm, Taf4,fra, CG7056, Ptp69D, CG4328, MED24, pum, Adf1, HLHm7, fd3F, sar1, ttk, Dlic2, sqz, ct, wts, trh, Tm1, Ptx1, ci, pros, acj6, put, Iswi, chm, mbf1, sens, dpn, fry, HmgD, Tab2, shot, CadN, gft, ss, Dp, Actbeta, Rho1, E(bx), Nrg, Su(z)12* |
| Larval locomotory behaviour^a^ | *Shab, Gad1, sbb, npf, EcR, Fmr1, unc-104, for, Sh, Adf1, syt, drpr, ppk, dpn, Tbh* |
| Memory^a^ | *dnc, Fas2, yu, N, for, shi, CaMKII, cer, pum, Adf1, drl, klg, rut, sra, scb, Nmdar1, sbr, Pka-C1, eas, Tbh, Nf1, drk* |
| Learning^a^ | *dnc, rogdi, Fas2, ltd, Src64B, pigeon, pxb, dikar, cher, rho, 14-3-3zeta, sgg, shi, Adf1, drl, klg, rut, sra, scb, Nmdar1, Pka-C1, Fas3, mol, gp210, eas, shn, mura, Nf1, drk* |
| Implicated in jump-reflex habituation^b^ | *eag, dnc, rut, for, Hk, Sh, slow* |
| Orthologs of genes misexpressed in *Ehmt1* mutant mouse brain^c, d^ | *GstS1^e^, stumps, Nc, CG31146, Corin, dac, Mhc^e^, Lasp, kazachoc, Prestin, CG10444, CG10031, up, bt, LanB1, CG9701* |

**^a^**Official ontology term according to www.geneontology.org. ^b^From Engel and Wu, 2009. ^c^Mammalian-to-fly orthology refers to one-to-one or many-to-one orthology. ^d^Schaefer *et al.*, 2009.^e^Fly genes represented by more than one mouse counterpart in Schaefer *et al.*, 2009.
