# Supplementary material for: Epigenetic Regulation of Learning and Memory by Drosophila EHMT/G9a
Source: PLoS Biol. 2011 Jan 4;9(1):e1000569. doi: 10.1371/journal.pbio.1000569 (PMC3014924; doi:10.1371/journal.pbio.1000569)
Supplement: Text S1 — Supplementary Materials, Methods, and Results. (0.09 MB DOC) [file pbio.1000569.s012.doc]

**Supplemental Text**

**Fly Stocks**

The following fly stocks were obtained from the Bloomington *Drosophila* stock center (Indiana University): *y^1^, EHMT^KG01242^* (FBst0013414); *w*; ry^506^,Sb^1^,P[Δ2-3]99B/TM6B,Tb^1^* (FBst0001798); *477-Gal4, UAS-memGFP* (FBst0008746); *109(2)80-Gal4, UAS-memGFP* (FBst0008768); *elav-Gal4/CyO* (FBst0008765); *7B-Gal4* (FBst0007365); *hs-Gal4*(FBst0002077).

For the creation of *EHMT* deletions *y^1^,EHMT^KG01242^* females were crossed to *w; ry^506^,Sb^1^,P[Δ2-3]99B/TM6B,Tb^1^* males in order to induce mobilization of the *KG01242* p-element insertion, which has a *y^+^* marker [1]. Male progeny with the genotype *y^1^,EHMT^KG01242^/Y*; *ry^506^,Sb^1^,P[Δ2-3]99B/+* were crossed to *C(1)DX, y^1^, f^1^/Y* females and male progeny with an excision of *KG01242* were isolated based on the absence of the *y^+^* marker. 80 independent excision lines were established and analyzed by PCR. *EHMT^+^*, *EHMT^DD1^* and *EHMT^DD2^* PCR products were sequenced to determine the excision sites.

For the creation of *UAS-EHMT* flies the cDNA *LD10243* was obtained from the Drosophila Genomic Resource Center (Indiana University) and cloned into the *P[UAST]* [2] vector by the Genescript Corporation. After conformation of the clone by sequencing it was used by BestGene Incorporated in the creation of transgenic flies.

**Western Blotting**

Protein samples were run on a two layer gel with a 6% layer for resolution of large proteins and a 10% layer for resolution of smaller proteins including the α-tubulin loading control. Blots were first probed with rabbit-anti-EHMT antibodies [3] at a 1/500 dilution and visualized using a goat-anti-rabbit-HRP antibody (Invitrogen) and the Supersignal West Pico Chemiluminescent Substrate (Pierce). As a control for equal loading of samples the same blot was probed with a rat-anti-α-tubulin antibody (AbD Serotec) at a 1/1000 dilution and visualized using a goat-ant-rati-IRdye800 antibody (Invitrogen) on an Odyssey infrared imager (LI-COR Biosciences).

**Quantitative real-time PCR**

The gene *CG3038* is positioned very close to the site of deletions in EHMT mutants. We therefore used quantitative real-time PCR (qPCR) to compare *CG3038* mRNA levels in *EHMT*^+^ and *EHMT^DD1^*. Extraction of mRNA from larvae and qPCR were performed as described [4], with the following exceptions. RNA was subjected to an extra DNAse treatment using the DNA-free kit (Ambion) before cDNA synthesis. *β’cop*, *eIF2b-γ*, and *RpII140* were used as reference genes and the average relative expression obtained using the three reference genes is presented. No difference in CG3038 mRAN levels was observed between EHMT mutant and wild type larvae (**Figure S2**).

**EHMT mutants have normal gross mushroom body morphology**

Mushroom bodies were visualized using the *7B-Gal4* [5] driver to express *UAS-memGFP*. Males of the genotypes *EHMT*^+^*; UAS-memGFP/+; 7B-Gal4/+*, *EHMT^DD1^; UAS-memGFP/+; 7B-Gal4/+*, and *EHMT^DD2^; UAS-memGFP/+; 7B-Gal4/+* were selected for dissection of brains. Several brains were analyzed from each genotype and no gross abnormalities in mushroom body structure, such as absence or deformation of lobes or midline crossing, were observed. Representative images are shown in **Figures S3a** and **S3b**.

**EHMT mutants have normal morphology of the larval neuromuscular junction**

For evaluation of larval neuromusclular junctions (NMJs) open-book preparations were performed as described [6] and immunolabeled with anti-DLG. Microscopic examination of NMJs revealed no noticeable difference in NMJ size or structure between *EHMT*^+^, *EHMT^DD1^*, and *EHMT^DD2^* (**Figures S3c** and **S3d**). This observation was confirmed by quantitative analysis of NMJ area using Image J, which revealed no difference in synapse area between *EHMT*^+^, *EHMT^DD1^*, and *EHMT^DD2^* (**Figure S3e**). Quantitative analysis of button number by manually counting under the microscope did reveal a decrease in the mean for *EHMT^DD2^* (**Figure S3f**). However, this difference was small (4 buttons less in *EHMT^DD2^*) and was not seen in *EHMT^DD1^*.

**Electroretinograms**

Electroretinograms were performed as described [7]. The average of 5 recordings from one fly is presented. Recordings were taken from 5 flies per genotype. No abnormalities were observed in electroretinograms of *EHMT* mutant flies (**Figure S3g**).

**Analysis of type 4 md neurons**

Images of type 4 md neurons were acquired from a region of 375 X 375 µm between the dentical belts of the abdominal segments 3-5 of wandering third instar larvae. Each larval hemi segment contains four clusters of md neurons (ventral, ventral’, lateral and dorsal), which are morphologically characterized and are positioned in a highly stereotypical pattern [8]. In this study we have analyzed the morphology of the solitary type 4 md neuron in the ventral cluster called vdaB (ventral dendritic arborization neuron B). Microscopic images were captured using a 40X lens. The field of view was always confined by the dentical belts, on the left (anterior) and right (posterior) margin of the image, with the cell body of the vdaB neuron positioned approximately in the middle of the field of view from top to bottom. Dendrite ends were counted manually from stacked images for the entire field of view using Image J.

**Analysis of larval locomotory behaviour**

Adult flies were allowed to lay eggs on grape juice agar over night. The following morning hatched larvae were removed from egg collections. After four hours, newly hatched larvae were transferred to culture dishes. Assays were performed with foraging third instar larvae at 96±2 hours post egg-hatching and no differences in developmental timing were observed between the different genotypes. Larvae were gently rinsed and transferred to the center of a 8.8 cm diameter test field containing a thin layer of yeast paste where they were allowed to crawl for 5 minutes. Their tracks, visible in the yeast paste, were traced and scanned for digital analysis.

**EHMT mutants display normal phototaxis and negative geotaxis behaviors**

In order to determine the role of *EHMT* in fly behavior we have tested *EHMT* mutants in several established behavioral paradigms including phototaxis and negative geotaxis.

The fast phototaxis assay is based on the flies natural tendency to move towards a light source and was performed as first described [9]. A countercurrent apparatus was used to fractionate a fly population amongst six tubes according to visual activity [9,10]. Flies were banged down to the bottom of a tube and the apparatus was laid down horizontally for 15 seconds, allowing the flies to move towards a light source in an otherwise dark area. Only flies with positive phototactic behavior reach the next tube. A phototaxis index (PI) was calculated based on the proportion of flies in each tube, the lowest score being 1 and the highest being 6 (PI=(∑i*N_i_)/N, where N is the number of flies, i is the tube number, and N_i_ is the number of flies in the ith tube). Three independent tests were performed with 40-50 seven day old flies from *EHMT*^+^, *EHMT^DD1^*, and *EHMT^DD2^* (3 X 40-50 = 120-150 flies tested per genotype). The average phototaxis index was not different between the three strains, which all showed a positive response to light (PI between 5 and 6) (**Figure S4a**). This shows that these strains have normal vision, locomotive ability, response to banging and retain their innate behavior to move towards light.

Flies have a natural tendency to climb up the side of a vial after being knocked down to the bottom called negative geotaxis. This behavior has been used to measure locomotive ability in fly models for neurodegenerative disease [11]. We assayed negative geotaxis in 7 day old flies using an established graded method to calculate a climbing index (CI)[12]. Flies that do not climb have a CI of 1 and flies who climb to the top of the gradient have a CI of 5. We found no difference in the climbing index of *EHMT*^+^, *EHMT^DD1^* and *EHMT^DD2^* which all had a mean CI close to 5 (**Figure S4b**). This shows that these strains have normal locomotive ability, response to banging, and retain their innate negative geotaxis behavior.

**Light-off jump reflex habituation**

Flies were collected at eclosion making sure that the females were virgin. They were kept in groups of 20 in small food vials for 3 to 5 days, and then tested in the behavior assay. In the high-throughput light-off jump reflex habituation system 16 flies were tested in parallel. They were kept in 16 individual semi-transparent plastic chambers in which they received 100 light-off stimuli with a 1 second time interval between each stimulus (the inter trial interval). Their jump responses were monitored by a 16-unit microphone-amplifier system, detecting the noise of the short flight following the jumps. These audio signals then were analyzed by a custom made LabView software (National Instruments). A response was recorded if the fly jumped during or within 300 milliseconds after a 15 millisecond light-off stimulus. All genotypes had a high initial jump response, which decreased upon repeated presentation of the stimulus (habituation). Flies were deemed to have habituated when a failure to jump occurred in five consecutive trials (no-jump criterion). Habituation was scored as the number of trials (light-offs) required to reach the no-jump criterion (Trials To Criterion, TTC). We calculated the mean Trials To Criterion for 16 flies (of same genotype) tested in parallel and repeated this with 6 independent groups of 16 (6 means x 16 = 96 flies were tested for each genotype). The mean and standard error of the 6 means was used as a basis for statistical comparison.

**Courtship Conditioning**

Male flies of the genotypes *EHMT^+^*, *EHMT^DD1^*, *EHMT^DD2^*, *EHMT^+^;elav-Gal4/UAS-EHMT*, *EHMT^DD2^;elav-Gal4/UAS-EHMT*, *EHMT^+^;7B-Gal4/UAS-EHMT*, *EHMT^DD2^;7B-Gal4/UAS-EHMT, EHMT^+^;hs-Gal4/+;UAS-EHMT/+*, *EHMT^DD2^;hs-Gal4/+;UAS-EHMT/+* were tested for learning and/or memory in the courtship conditioning assay as previously described [13]. Males were collected at eclosion and kept in isolation for 4 days before training, in which male flies were paired with a single premated female in a food chamber for 1 hour (learning and short term memory; **Figures 5d** and **5e**) or 7 hours (long term memory and short term memory in the *hs-Gal4* rescue experiment; **Figures 5d** and **5f**). Courtship activity towards a single premated Oregon R female was assayed in a 1 cm diameter chamber either immediately after training (learning) 30-60 minutes after training (short term memory) or 24 hours after training (long term memory) and was quantified manually from videos. For each genotype and condition (naïve or trained) a total of 45-55 flies were assayed in independent groups of 10-14 over the course of three to four days. Independent Learning Indexes were calculated for each genotype on each day and the mean Learning Index over the course of 4 days was used as a basis for statistical comparison.

**Comparison of EHMT protein levels in the adult brain upon expression with elav-Gal4 and 7B-Gal4.**

Adult brains were dissected and stained with anti-EHMT and anti-dac, which labels the nuclei of Kenyon cells, which form the mushroom body. Images were obtained using identical settings on a Lieca confocal microscope (Leica Microsystems). The merged images show that *UAS-EHMT* expression is highest in the *EHMT*^+^ background with *7B-Gal4*, since the colour overlay is more green than in the other genotypes, which are more magenta and white (overlay of magenta and green produces white)(**Figure S6a**). We quantified this effect using image J to measure intensity levels in the area contained by dac positive cells. This quantification reflects what is seen in the merged images (**Figure S6b**).

**ChIP-seq**

Third instar larvae were homogenized in PBS followed by crosslinking with 1% formaldehyde. Crosslinked chromatin was fragmented by sonicating four times for eight minutes (high power, 30 seconds on/off) with a Bioruptor (Diagenode). Chromatin immunoprecipitation was performed with anti-H3K9me2 antibodies (07-441, Upstate) and Prot A/G beads (Santa Cruz) were used to capture antibody bound chromatin. End repair was performed on 10-20 ng of enriched ChIPed DNA (measured by Qubit fluorometer) using the Quant-iT dsDNA HS Assay Kit from (Invitrogen, Q32851). Adaptors were ligated to DNA fragments, which were subsequently size selected (~300 bp). The adapter-modified DNA fragments were subjected to limited PCR amplification (14 cycles) and quality control was made by qPCR, as well as by running the PCR products on a Bioanalyzer (BioRad). Finally, cluster generation and sequencing-by-synthesis (36 bp) was performed using the Illumina Genome Analyzer IIx according to standard protocols of the manufacturer (Illumina). The image files generated by the Genome Analyzer were processed to extract DNA sequence data. Sequences were aligned to the *Drosophila* genome using the Illumina Analysis Pipeline allowing one mismatch. Only the tags uniquely aligning to the genome were considered for further analysis. The 36 bp sequence reads were directionally extended to 300 bp, corresponding to the length of the original fragments used for sequencing.

**Supplemental** **References**

1. Spradling AC, Stern D, Beaton A, Rhem EJ, Laverty T et al. (1999) The Berkeley Drosophila Genome Project gene disruption project: Single P-element insertions mutating 25% of vital Drosophila genes. Genetics 153: 135-177.

2. Brand AH, Perrimon N (1993) Targeted gene expression as a means of altering cell fates and generating dominant phenotypes. Development 118: 401-415.

3. Stabell M, Eskeland R, Bjorkmo M, Larsson J, Aalen RB et al. (2006) The Drosophila G9a gene encodes a multi-catalytic histone methyltransferase required for normal development. Nucleic Acids Res 34: 4609-4621.

4. Mukhopadhyay A, Kramer JM, Merkx G, Lugtenberg D, Smeets DF et al. (2010) CDK19 is disrupted in a female patient with bilateral congenital retinal folds, microcephaly and mild mental retardation. Hum Genet 128: 281-291.

5. Ferveur JF, Savarit F, O'Kane CJ, Sureau G, Greenspan RJ et al. (1997) Genetic feminization of pheromones and its behavioral consequences in Drosophila males. Science 276: 1555-1558.

6. Schenck A, Bardoni B, Langmann C, Harden N, Mandel JL et al. (2003) CYFIP/Sra-1 controls neuronal connectivity in Drosophila and links the Rac1 GTPase pathway to the fragile X protein. Neuron 38: 887-898.

7. Verstreken P, Koh TW, Schulze KL, Zhai RG, Hiesinger PR et al. (2003) Synaptojanin is recruited by endophilin to promote synaptic vesicle uncoating. Neuron 40: 733-748.

8. Grueber WB, Jan LY, Jan YN (2002) Tiling of the Drosophila epidermis by multidendritic sensory neurons. Development 129: 2867-2878.

9. Benzer S (1967) Behavioral mutants of Drosophila isolated by countercurrent distribution. Proc Natl Acad Sci U S A 58: 1112-1119.

10. Galy A, Roux MJ, Sahel JA, Leveillard T, Giangrande A (2005) Rhodopsin maturation defects induce photoreceptor death by apoptosis: a fly model for RhodopsinPro23His human retinitis pigmentosa. Hum Mol Genet 14: 2547-2557.

11. Feany MB, Bender WW (2000) A Drosophila model of Parkinson's disease. Nature 404: 394-398.

12. Todd AM, Staveley BE (2004) Novel assay and analysis for measuring climbing ability in Drosophila. Drosoph Inf Serv 87: 101-107.

13. Keleman K, Kruttner S, Alenius M, Dickson BJ (2007) Function of the Drosophila CPEB protein Orb2 in long-term courtship memory. Nat Neurosci 10: 1587-1593.
